# Risk factors for nosocomial infections in ECMO patients: a systematic review and meta-analysis

**DOI:** 10.3389/fpubh.2026.1820017

**Published:** 2026-06-11

**Authors:** Jia Duan, Lei Yang, YanFang Huang, Duo Li, Dan Liu

**Affiliations:** 1 Department of Intensive Care Medicine, Neijiang Hospital of Traditional Chinese Medicine, Neijiang, Sichuan, China; 2 Department of Geriatrics, Neijiang First People's Hospital, Neijiang, Sichuan, China; 3 Department of Critical Care Medicine, Affiliated Hospital of Southwest Medical University, Luzhou, Sichuan, China; 4 Department of Pulmonary and Critical Care Medicine, Affiliated Hospital of Southwest Medical University, Luzhou, Sichuan, China; 5 Department of Infection Management, Affiliated Hospital of Southwest Medical University, Luzhou, Sichuan, China

**Keywords:** extracorporeal membrane oxygenation, meta-analysis, nosocomial infections, risk factors, system review

## Abstract

**Background:**

Nosocomial infection (NSI) is a serious complication in extracorporeal membrane oxygenation (ECMO) patients, yet reported risk factors remain inconsistent. A systematic synthesis is needed to identify key predictors.

**Methods:**

Following PRISMA guidelines, this meta-analysis (PROSPERO: CRD420251161290) searched five databases for studies reporting multivariate risk factors for NSI in adult ECMO patients. Study selection, data extraction, and quality assessment (Newcastle-Ottawa Scale) were performed independently by two reviewers. Pooled odds ratios (ORs) were calculated with random-effects models; heterogeneity (I^2^ ≥ 50%) was explored via meta-regression and subgroup analyses.

**Results:**

Thirty-four observational studies (≈8,901 patients) were included. Significant risk factors were: immunosuppression (OR 2.21, 95% CI 1.27–3.85), inter-hospital transport (2.24, 1.04–4.80), pre-ECMO infection (2.54, 2.03–3.18), higher SOFA score (per point: 1.13, 1.03–1.24), prolonged ECMO duration (per day: 1.10, 1.07–1.14), continuous renal replacement therapy (2.18, 1.57–3.02), and catheter intubation (1.24, 1.02–1.51). ECMO duration showed high heterogeneity (I^2^ = 89.9%), largely driven by small-study effects. Age, body mass index, diabetes mellitus, and hypertension were not significantly associated.

**Conclusion:**

NSI in ECMO patients arises from an interplay of baseline characteristics, illness severity, and invasive support duration/intensity. A stratified, risk-factor-based prevention strategy is warranted. Given the predominantly retrospective evidence, prospective studies are required for validation.

**Systematic review registration:**

https://www.crd.york.ac.uk/PROSPERO/view/CRD420251161290, Identifier: CRD420251161290.

## Introduction

Extracorporeal membrane oxygenation (ECMO) has become an indispensable ultimate life support modality for critically ill patients with severe respiratory failure, cardiogenic shock, and cardiac arrest. ECMO is a form of extracorporeal life support technology that functions by draining venous blood from the patient, passing it through an oxygenator for gas exchange, and then returning the oxygenated blood to the patient’s body. However, nosocomial infections (NSI), as one of the most common and severe complications during ECMO therapy, remain a core challenge affecting patient outcomes (1–3). Studies have reported that the incidence of ECMO-related infections varies widely across single-center studies, ranging from 4.1 to 85.4%, with the most recent meta-analyses providing more precise pooled estimates. Patients with NSI have a 16 and 20% reduction in ECMO survival and overall survival, respectively, while the risk of in-hospital death is relatively increased by 37% (4).

Beyond the direct impact on mortality, the epidemiological characteristics of ECMO-related infections have undergone significant changes over the past two decades. Early data from the Extracorporeal Life Support Organization (ELSO) registry indicated that bloodstream infections (BSI) and Gram-positive bacteria (e.g., coagulase-negative staphylococci) were once the predominant infection types and pathogens (3). However, ventilator-associated pneumonia (VAP) has now replaced BSI as the most common type of NSI, while Gram-negative bacteria such as *Acinetobacter baumannii*, *Klebsiella pneumoniae*, and *Pseudomonas aeruginosa—*particularly multidrug-resistant (MDR) strains—have become the primary causative pathogens. This shift in etiology is closely related to antimicrobial pressure, changes in the ICU ecological environment, and evolving patient population characteristics, posing more severe challenges to anti-infective therapy.

Regarding risk factors, existing meta-analyses reveal a landscape of both consensus and controversy. Multiple studies consistently confirm that prolonged ECMO support duration is the most robust risk factor for NSI, increasing the infection risk several-fold (4–6). A study from a tertiary hospital demonstrated that ECMO treatment duration ≥10 days was an independent risk factor for ECMO-related nosocomial infections. In addition to ECMO duration, mechanical ventilation time, ICU length of stay, use of continuous renal replacement therapy (CRRT), red blood cell transfusion, immunosuppressive status, and VA-ECMO mode are all significant risk factors. However, for other factors such as patient age, sex, or obesity (BMI), conclusions from different studies and even different meta-analyses remain contradictory (2, 4, 6, 7).

The diagnostic criteria for ECMO-related infections still lack a gold standard, and the interpretation of risk factors goes far beyond simple association analysis; potential confounding biases must be carefully considered. For example, the difference in infection risk between VA-ECMO and VV-ECMO modes is likely rooted in fundamental differences in the underlying diseases of supported patients (cardiogenic shock vs. respiratory failure), cannulation strategies (involving arterial access vs. venous-only), and disease severity (8). Furthermore, the diagnosis of ECMO-related infections is challenging, as clinical signs such as temperature changes may be difficult to interpret due to the influence of the ECMO circuit’s heat exchanger settings.

Although several meta-analyses have preliminarily explored the above issues, they either failed to quantify the specific risk factors for different types of infections (e.g., VAP vs. BSI) or could not effectively adjust for biases arising from the substantial heterogeneity in diagnostic criteria and patient populations during pooled analyses (2, 4, 6). Therefore, it is necessary to conduct a new meta-analysis focusing on risk factors, systematically addressing the contradictions in existing evidence and clarifying key driving factors by pooling larger sample sizes and implementing stricter subgroup analyses. The present study aims to achieve this goal by integrating the latest evidence, thereby providing more reliable and in-depth evidence-based support for constructing precise infection risk prediction models and formulating individualized prevention strategies in clinical practice.

This study aims to compare the impact of different modifiable factors on nosocomial infections in ECMO patients through meta-analysis, providing evidence-based guidance for clinical staff to develop targeted infection prevention strategies, ultimately reducing the incidence and associated mortality of nosocomial infections in ECMO patients.

## Methods

### Search strategy and selection criteria

In this systematic review and meta-analysis, we adhered to the PRISMA reporting guidelines (9). This study has been registered in PROSPERO, registration number CRD420251161290.

Population-based, case–control, randomized controlled/cohort studies (i.e., regional, national, or multicenter studies) reporting risk factors for infection in adult patients supported by ECMO were eligible for inclusion. When a study included participants not fully meeting this criterion, but reported results for a pre-specified, eligible subgroup (e.g., an adult subgroup) with complete effect estimates (HR, OR, or RR with 95% CI), we extracted the subgroup-specific effect size rather than the overall study result. If both overall and subgroup estimates were reported, only the eligible subgroup was included to avoid double counting. Articles were required to present results from multivariate regression analysis to enhance result credibility. Unpublished papers, preprints, editorials, commentaries, letters, case reports, books, and studies in non-human subjects or non-English languages were excluded. We also excluded meta-analyses and systematic review papers but assessed the reference lists of relevant reviews to screen for potentially eligible studies. Five electronic databases (PubMed, Web of Science, Embase, Cochrane Library, and EBSCO) were searched. Reference lists of included studies and available reviews were examined to identify further eligible studies. The search strategy used keywords “Risk factors,” “extracorporeal membrane oxygenation,” and “infection” in titles and abstracts, and as MeSH terms, employing a comprehensive search string to identify eligible studies. The search was limited to papers retrievable from database inception until September 24, 2025 (Supplementary Table S1).

### Data extraction and quality assessment

Two researchers (Jia Duan, Lei Yang) independently conducted screening, initially based on titles and abstracts, followed by full-text review when appropriate, to determine eligibility according to inclusion criteria. Any disagreements regarding inclusion were resolved through consensus or discussion with other review authors (Dan Liu, Duo Li). Two authors (Jia Duan, Lei Yang) independently extracted data from the finally selected studies. When duplicate data from the same source population were identified, data from the earlier study were removed. The following data and information were extracted from each eligible study using a pre-tested data extraction form: first author’s surname, publication year, country, study duration, sample size, number of infected patients, gender, study design type, various types of NSI, ECMO type, effect estimates (odds ratios, ORs) and confidence intervals (CIs) from multivariate regression analyses for infection factors. Included studies generally defined ECMO-associated nosocomial infection (referring to local or internationally accepted diagnostic criteria for nosocomial infection) as: infections occurring between 24 h after ECMO initiation and 48 h after ECMO cessation were considered ECMO-associated nosocomial infections, excluding pre-existing infections before ECMO use. Various types of NSI included bloodstream infection (BSI), respiratory tract infection (RTI), urinary tract infection (UTI), surgical site infection (SSI), cannulation site infection (CSI), and ventilator-associated pneumonia (VAP).

Two authors (Jia Duan and Lei Yang) assessed the quality of the literature using the Newcastle-Ottawa Scale (NOS) (10) criteria. The NOS includes three parts: selection of study groups, comparability of groups, and ascertainment of exposure/outcome, with a maximum score of 9. Scores of 7–9 indicate high quality, 5–6 moderate quality, and ≤4 low quality. Considering potential bias in retrospective studies, we required an NOS score ≥6 for inclusion in the analysis. All disagreements were resolved after discussion with other authors (YanFang Huang, Dan Liu, and Duo Li).

We explored publication bias by generating funnel plots for comparisons with ≥10 studies, visually assessing funnel plot asymmetry. For comparisons with ≥5 studies, we reported the *p*-value from Egger’s test. The stability of conclusions was primarily assessed using the leave-one-out method for all studies.

### Data analysis

Pooled incidence rates of ECMO-associated nosocomial infection for different risk factors and their 95% CIs were calculated using random-effects models. Included studies reported associations using hazard ratios (HRs), odds ratios (ORs), or risk ratios (RRs). Because these effect measures differ in definition and underlying assumptions, no direct pooling of HRs, ORs, and RRs was performed. Instead, separate meta-analyses were conducted for each effect measure type when feasible. The synthesis strategy was as follows:(1) When two or more studies reported the same type of effect measure (e.g., OR), a random-effects meta-analysis (DerSimonian and Laird method) was used to calculate a pooled estimate with 95% confidence interval.(2) When a particular effect measure was reported by only a single study, quantitative synthesis was not possible; the results of that study were summarized descriptively (point estimate, 95% CI, and direction of association).(3) The overall evidence was then presented by combining the pooled estimates with descriptive summaries, i.e., a joint presentation of meta-analytic and narrative results.

Subgroup analyses were performed when necessary. The choice of model was based on I^2^ statistics: a fixed-effects model was used for I^2^ < 50%, and a random-effects model for I^2^ ≥ 50%. Based on expectations, we anticipated that a random-effects model would be used for most analyses, as the exposures were unlikely to be truly identical across included studies. ECMO-associated nosocomial infection factors were categorized into modifiable and non-modifiable factors. When fewer than two studies were available for a specific comparison, meta-analysis could not be performed, and only statistically significant risk factors were described. Forest plots for non-significant factors are presented in the appendix. Statistical analysis was performed using STATA version 14.0.

## Results

### Study characteristics

**Figure 1:**
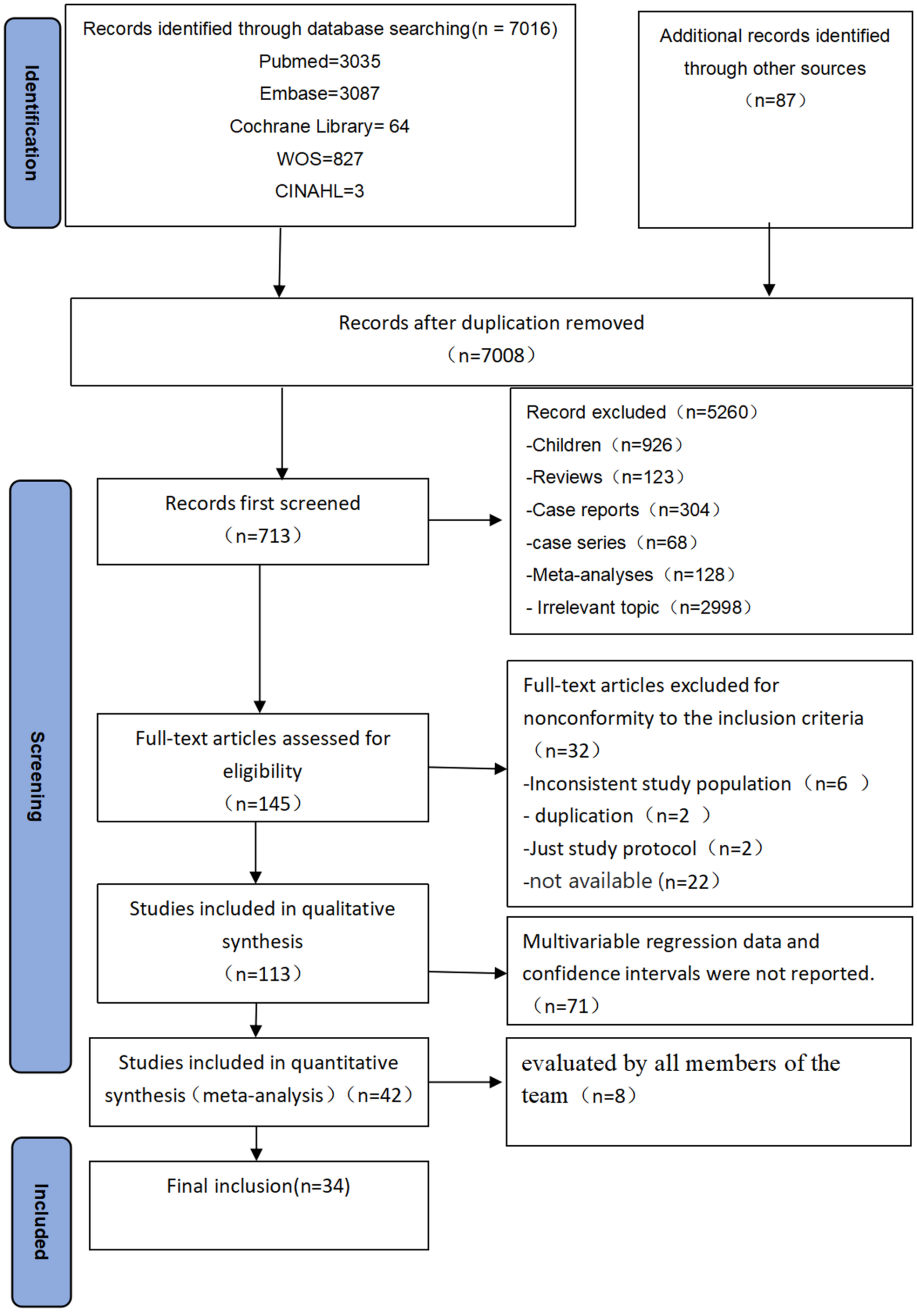
Flow diagram.

A total of 7,103 articles were identified. After removing 95 duplicates, the remaining 7,008 articles were screened based on titles and abstracts: ineligible articles included case reports, book chapters, comments, editorials, non-English manuscripts (or those without available English translation), non-human studies, reviews, or duplicate studies. Full-text screening was performed on 145 articles, ultimately including 113 articles for risk factor assessment. Among these, 42 studies provided data for etiology and risk factor analysis. After being evaluated by all members of the team, 34 articles were finally included. The literature screening flowchart is shown in Figure 1.

### Basic characteristics of included studies

Among the 34 included studies (6–40), the time span ranged from 2001 to 2024, covering 15 countries/regions including China (including Taiwan), Italy, France, South Korea, the United States, and Australia. The study types were predominantly retrospective (29 studies), with 5 prospective studies. The total number of included patients was approximately 8,901. The total sample sizes varied greatly, the basic characteristics of the included studies are shown in Table 1.

**Table 1:** Clinical features.

Author	Year	Country/Region	Study period	Study Type	Age (Mean ± SD/IQR)	Sample Size	Male	Female	ECMO mode	Infection types
Aubron C	2013	Australia	2005.01–2011.06	Prospective	48.5(37–57) 45(31–56)	146	107	39	VA:96; VV:50	BSI: 24
Grasselli G	2017	Italy	2010.1–2015.11	Retrospective	48.5(37–56)	92	58	34	VV: 80; Other: 12	VAP: 32; UTI:8; BSI:5
Bachleda T	2015	Austria	2008–2012	Retrospective		134	NR	NR	VA	NR
Hsu M-S	2009	Taiwan, China	2001.7–2007.6	Retrospective	42.1 ± 13.9 52.2 ± 17.3	114	77	37	VA: 98; VV: 16	VAP:4; SSI:4; BSI:3
Sun H-Y	2010	Taiwan, China	1996.1–2007.12	Retrospective	47 ± 15 52 ± 17	334	NR	NR	VV: 57; VA: 265	BSI:38; SSI:6; VAP:4
Vogel A M	2011	Multicenter	1987–2009	Retrospective	45.3 ± 16.9 43.5 ± 16.4	2,995	NR	NR	NR	NSI:3,960
Schmidt M	2012	Paris	2003.1–2009.12	Retrospective	49.5 ± 16.9	220	147	73	VA	NSI:142
					47.9 ± 15.1					VAP: Most common type
Bougle A	2018	France	2013.1–2014.12	Retrospective	64.3 ± 11.4 57.4 ± 14.9	152	112	40	VA	VAP:85
Kim G S	2017	Korea	2011.1–2015.12	Retrospective	58.5 ± 12.5 61.3 ± 14.9	61	40	21	VA: 58; VV: 3	VAP:9; BSI:9
Li Z-J	2021	China	2013.1–2019.12	Retrospective	51.1 ± 13.2 47.4 ± 15.1	56	33	23	VA: 52; VV: 4	VAP: 18; BSI:3
Kang J	2025	China	2021.1–2024.3	Retrospective	53.3 ± 18.3 54.2 ± 17.2	59	36	23	NR	BSI:12
Na S J	2018	Korea	2012.1–2016.12	Retrospective	56(50–65) 60(51–68)	121	88	33	VV	BSI:21
Carelli S	2023	Italy	2020.3–2022.3	Prospective	53(43–60) 54(51–60)	68	NR	NR	NR	NR
Wang J-R	2020	China	2013.1–2019.8	Retrospective	44(21–67) 40(18–77)	69	43	26	VA: 58; VV: 11	BSI: 19
Allou N	2018	France	2010.1–2016.12	Retrospective	56(42–65) 52(43–63)	220	NR	NR	VA: 151; Other: 69	CRI: 39
Yeo H J	2020	Korea	2017.3–2019.8	Prospective	59.4 ± 15.2 56.7 ± 13.4	192	123	69	VA: 86; VV: 106	CC: 11
Lee E H	2022	Korea	2015.1–2021.5	Retrospective	58.8 ± 12.9 55.5 ± 14.9	480	314	166	VA: 244; VV: 130; VAV: 106	BSI: 112
Hao T	2024	China	2011.1–2019.12	Retrospective	52(39–59) 47(33–56)	174	128	46	VA: 77; VV: 97	NSI: 46
Wang J	2021	China	2012.1–2017.12	Retrospective	57.1 ± 12.4 57.30 ± 11.52	322	239	83	VA	NSI: 131
Juthani B K	2018	America	2012.1–2015.7	Retrospective	51.2 ± 14.3 52.6 ± 16.2	100	59	41	VV: 64; VA: 36	NSI: 26
Manerikar A	2022	America	2015.1–2019.2	Retrospective	45.5 ± 14.7 48.1 ± 15.7	61	45	16	VV	BSI
Rodriguez-Goncer I	2018	England	2012.1–2016.12	Retrospective	44.3(16.4–73.4)	134	88	46	VV	IPF: 10
Massart N	2023	France	2010.1–2021.12	Retrospective	51 (39–61) 51 (39–61)	241	NR	NR	VV	VAP: 107; BSI: 40
Kutleša M	2017	Croatia	2009.10–2016.6	Prospective	54.0 (42.0–64.0) 49.0 (33.0–60.0)					
Pinna S M	2023	Spain	2013.7–2019.3	Retrospective	55 (50.6–61.1) 59 (50–64.5)	69	57	12	VA	NSI: 29; BSI: 3; VAP:19
Ko R-E	2020	Korea	2010.1–2018.12	Retrospective	67 (52.5–73.0) 60.0 (50.0–69.5)	150	112	38	VA	NSI: 35
Xu W	2022	China	2011.1–2020.9	Retrospective	57.3 ± 13.9 48.8 ± 15.5	79	52	27	VV: 49; VA: 30	NSI: 42; VAP:30; BSI:19
Kuo L-P	2023	Taiwan, China	2002.1–2022.1	Retrospective	60.1(47.9–69.6) 55.5(41.9–65.4)	105	NR	NR	VA	NSI: 57; VAP:70; BSI:17
Yang L	2022	China	2013.8–2019.3	Retrospective	51 ± 16	105	76	29	VV	BSI:23
Deng Q	2024	China	2015.5–2022.12	Retrospective		192	150	42	VA: 100; VV: 92	VAP: 107; BSI: 28; UTI: 5
Wang L	2023	China	2015.1–2021.10	Retrospective	57.8 ± 14.6 55.4 ± 17.2	196	138	58	VA: 130; VV: 66	NSI: 86; HAI: 59; BSI: 36; UTI: 10; SSTI: 5
R. R. Ling	2023	Multicenter				1,237	nr	nr	nr	NSI; 693
Marcus J E	2021	America	2012.5–2020.5	Retrospective	43 (33–60) 39 (31–53)	123	nr	nr	nr	BSI: Local 5% vs. Transport 22%; VAP: Local 18% vs. Transport 26%
Winiszewski H	2022	France	2017.10–2019.11	Prospective	61.5 (53–70) 62 (52.8–66.0)	100	nr	nr	VA: 89; VV: 11	CRI: 24

NR, Not Reported; VA, Venoarterial ECMO; VV, Venovenous ECMO; VAD, Ventricular Assist Device (likely used in conjunction with ECMO); VAV, Venoarteriovenous ECMO; VAP, Ventilator-Associated Pneumonia; UTI, Urinary Tract Infection; BSI, Bloodstream Infection; SSTI, Skin and Soft Tissue Infection; NSI, Nosocomial Infection; SSI, Surgical Site Infection; CRI, Catheter-Related Infection; HAI, Hospital-Acquired Infection; CC, Catheter Colonization; IPF, Invasive Pulmonary Aspergillosis.

### Quality assessment

The NOS quality assessment results showed that among the 34 included studies, NOS scores ranged from 6 to 9, with an average score of 7.58. According to common standards (≥7 indicating high quality), 82.4% were high-quality literature, with no low-quality (high risk of bias) studies. The inter-rater agreement for NOS scores was moderate (weighted Kappa = 0.56), 95% CI: (0.33, 0.78). The risk of bias assessment results for included studies are shown in Table 2.

**Table 2:** Pooled results of NOS quality assessment.

Study	Selection of study population				Comparability of groups		Outcome measurement			Total score
	1	2	3	4	5.1	5.2	6	7	8	
Aubron C	1	1	1	1	1	1	0	1	1	8
Grasselli G	1	1	1	1	1	1	1	0	1	8
Bachleda T	1	1	1	0	1	1	1	0	0	6
Hsu M-S	1	1	1	1	1	1	1	0	1	8
Sun H-Y	1	1	1	0	1	1	1	1	1	8
Vogel A M	1	1	1	0	1	1	1	0	0	6
Schmidt M	1	1	1	1	0	1	1	0	1	7
Bougle A	1	1	1	1	1	0	1	1	1	8
Kim G S	1	1	1	1	1	1	1	1	1	9
Li Z-J	1	1	1	1	1	1	1	0	1	8
Kang J	1	1	1	1	1	0	1	1	1	8
Na S J	1	1	1	1	1	1	1	1	1	9
Carelli S	1	1	1	1	1	1	1	0	1	8
Allou N	1	1	1	0	1	0	1	1	0	6
Yeo H J	1	1	1	0	1	0	1	1	1	7
Lee E H	1	1	1	0	1	0	1	1	1	7
Hao T	1	1	1	1	1	0	1	1	1	8
Wang J	1	1	1	1	1	0	1	1	1	8
Juthani B K	1	1	1	1	1	0	1	0	1	7
Manerikar A	1	1	1	1	1	0	1	1	1	8
Rodriguez-Goncer I	1	1	1	0	1	0	1	1	1	7
Massart N	1	1	1	1	1	1	1	1	1	9
Kutleša M	1	1	1	1	1	0	1	1	1	8
Pinna S M	1	1	1	1	0	0	1	1	1	7
Ko R-E	1	1	1	0	1	0	1	1	1	7
Xu W	1	1	1	0	1	0	1	1	1	7
Kuo L-P	1	1	1	0	0	0	1	1	1	6
Wang J-R	1	1	1	1	0	0	1	1	1	7
Yang L	1	1	1	1	0	1	1	1	1	8
Deng Q	1	1	1	1	1	1	1	1	1	9
Wang L	1	1	1	1	1	0	1	1	1	8
R. R. Ling	1	1	1	1	1	0	1	1	1	8
Marcus J E	1	1	1	0	1	1	1	0	1	7
Winiszewski H	1	1	1	0	1	1	1	1	1	8

1. Exposure group selection; 2. non-exposure group selection; 3. method for determining exposure; 4. here are no outcome events at the beginning of the study; 5.1 controlling major confounding factors; 5.2. controlling other confounding factors; 6. adequate evaluation; 7. adequate follow-up; 8. follow up integrity.

### Meta-analysis of nosocomial infection risk factors

Twenty-two risk factors were reported by more than two studies: 1. Immunosuppression; 2. Gender; 3. Transport; 4. Virus; 5. Comorbidities; 6. Infection; 7. Mechanical ventilation; 8. Duration to ECMO; 9. C-reactive protein; 10. Hospital Stay Days; 11. Lactate; 12. SAPS II; 13. Body Mass Index; 14. SOFA; 15. Hypertension; 16. CRRT; 17. Diabetes (T2DM); 18. Mode; 19. Age; 20. ECMO Duration; 21. Catheter Intubation; 22. Arterial Catheter. Supplementary information is provided in Supplementary Table S2. The infection criteria and primary pathogens are presented in Supplementary Table S3. Based on clinical logic, the factors were categorized into three groups for presentation: (1) Baseline characteristics and comorbidities; (2) Disease severity and laboratory indicators; and (3) ECMO and treatment related factors. The meta-analysis results are shown in Table 3.

**Table 3:** Summary of meta-analysis results.

Risk factor	Studies		Pooled OR(HR/RR) (95% CI)	I^2^ value	*p*-value (Overall effect)
	Inclusion	Analysis^#^			
Immunosuppression	**8**	**5**	**2.21 (1.27, 3.85)**	**47.30%**	**0.005**
Gender	5	3	2.20 (0.70, 6.93)	66.30%	0.177
Transport	**3**	**2**	**2.24 (1.04, 4.80)**	**0.00%**	**0.038**
Virus^@^	5	3	1.17 (0.33, 4.16)	79.40%	0.809
Comorbidities^$^	4				
Comorbidities	**Mechanical**	**3**	**1.99 (1.34, 2.97)**	**65.50%**	**0.055**
Comorbidities	**Pulmonary**	**2**	**2.63 (1.61, 4.30)**	0.00%	0.398
Comorbidities	**Metabolic**	**2**	**2.21 (1.77, 2.75)**	0.00%	0.564
Infection^&^	**4**	**4**	**2.54 (2.03, 3.18)**	0.00%	< 0.001
Mechanical ventilation	5	2(Continuous*)	1.56 (0.89, 2.75)	59.40%	0.118
		3(Binary*)	1.06 (0.81, 1.37)	87.30%	0.655
Duration to ECMO	2	2	1.00 (0.99, 1.00)	0.00%	0.46
C-reactive protein	**2**	**2**	**1.00 (1.00, 1.01)**	0.00%	0.106
Hospital Stay Days	3	3	0.99 (0.96, 1.02)	0.00%	0.509
Lactate	4	2	1.11 (0.81, 1.52)	86.00%	0.509
SAPS II	3	2	1.02 (0.99, 1.04)	57.60%	0.106
Body Mass Index	2	2	0.97 (0.79, 1.20)	89.50%	0.786
SOFA	**5**	**3**	**1.13 (1.03, 1.24)**	32.50%	0.011
Hypertension	4	2	1.20 (0.68, 2.13)	17.50%	0.53
		2(RR)	1.37 (0.99, 1.89)	0.00%	0.057
CRRT	**5**	**4**	**2.18 (1.57, 3.02)**	16.70%	< 0.001
Diabetes(T2DM)	6	2(HR)	1.09 (0.80, 1.50)	26.80%	0.574
		2	1.62 (0.65, 4.07)	58.60%	0.304
Mode	7				
	VVvsVA	2(HR)	0.45 (0.21, 0.93)	0.00%	0.031
	VVvsVA	5	1.61 (0.45, 5.81)	73.90%	0.464
	VAVvsVA	2	3.66 (0.70,19.15)	68.50%	0.126
Age	9	6	1.00 (0.99, 1.02)	60.30%	0.708
ECMO Duration	**20**	**18**	**1.10 (1.07, 1.14)**	76.90%	< 0.001
Catheter Intubation^	**2**	**2**	**1.24(1.02,1.51)**	0.00%	0.032

Categorical Variable: Immunosuppression; Gender; Transport; Virus; Comorbidities; Hypertension; CRRT; Diabetes(T2DM); Mode; Catheter Intubation; Mechanical ventilation (Binary). Continuous Variable: Duration to ECMO; ECMO Duration; Hospital Stay Days; Mechanical ventilation (Continuous): For each additional day; C-reactive protein: For every increase of 1 mg/L; Lactate: For every increase of 1 mmol/mL; SAPS II; SOFA: For every additional point; Body Mass Index: For every increase of 1 kg/m^2^; Age: For each additional year; #. HR/RR have been separately identified. *. Continuous: Continuous Variable. Binary: Categorical Variable; @. Virus: Admission due to viral infection. $. Comorbidities: 1. Mechanical complications included oxygenator failure; pump malfunction; heater dysfunction; clot of oxygenator, hemofilter, and other sites; connector crack; and so forth. 2. Pulmonary complications included pneumothorax and pulmonary hemorrhage. 3. Metabolic complications included blood glucose level<40 mg/dL or >240 mg/dL, serum pH <7.2 or >7.6, or serum bilirubin level >15 mg/dL. &. Infection: present prior to the initiation of extracorporeal membrane oxygenation (ECMO), but with a causative pathogen that differs from the target infection.^. Catheter Intubation: Various types of drainage tubes or invasive monitoring catheters that are left in place in the body, excluding central venous catheters.

### Baseline characteristics and comorbidities

**Figure 2:**
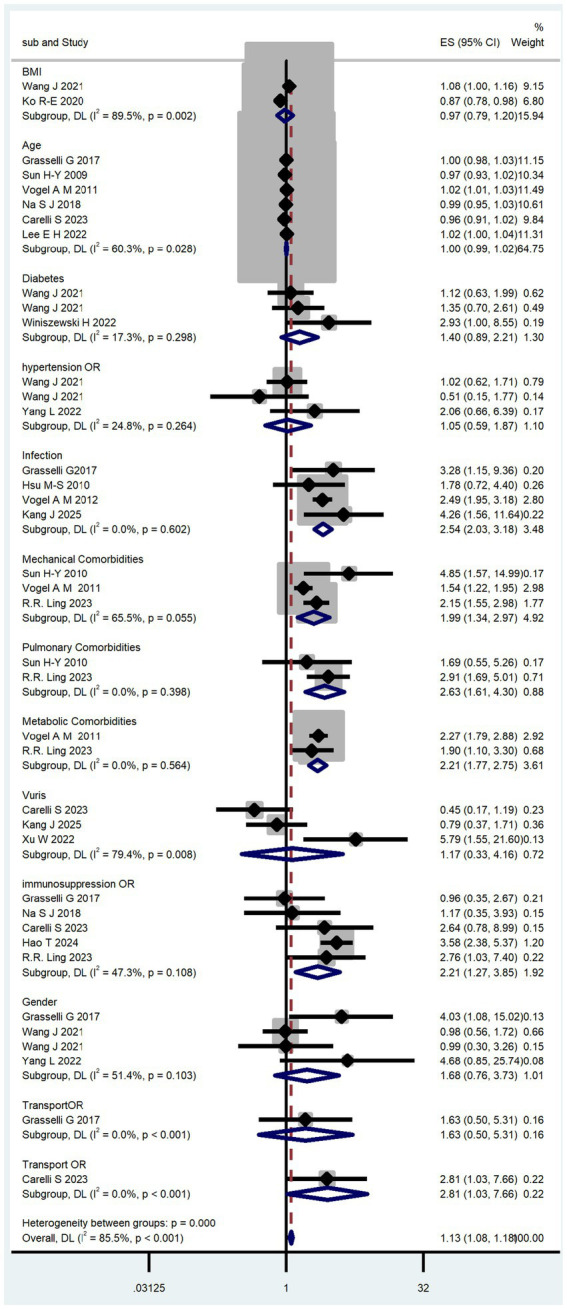
Forest plot summary of patient baseline characteristics and comorbidities.

In the category of baseline characteristics and comorbidities (Figure 2), immunosuppression was a significant and consistent risk factor for infection. The pooled odds ratio (OR) was 2.21 (95% CI 1.27–3.85, *p* = 0.005) with low heterogeneity (I^2^ = 47.3%). However, the pooled hazard ratio (HR) did not demonstrate an overall effect (HR = 1.34, 95% CI 0.32–5.60) and exhibited extremely high heterogeneity (I^2^ = 86%). This discrepancy primarily arose because the HR analysis included subgroups with strong effect direction heterogeneity, specifically the Invasive Pulmonary Aspergillosis (IPA) subgroup (HR = 10.75) and the NSI subgroup (HR < 1); the test for between-group heterogeneity was *p* = 0.001, confirming that stratification by infection type is justified. Three studies reported on inter-hospital transport; a pooled OR of 2.24 (95% CI 1.04–4.80) from two of these studies suggested that transport increases infection risk. Four studies examined the presence of comorbidities. Metabolic, mechanical, and pulmonary comorbidities all significantly increased infection risk, with pooled ORs of 2.21 (95% CI 1.77–2.75), 1.99 (95% CI 1.34–2.97), and 2.63 (95% CI 1.61–4.30), respectively. Pre-ECMO infection, reported in four studies, was a strong predictor (OR = 2.54, 95% CI 2.03–3.18, *p* < 0.001) with no heterogeneity (I^2^ = 0%). Age, diabetes, gender, and viral etiology did not reach statistical significance. The pooled OR for hypertension was not significant, and the pooled risk ratio (RR) also did not reach significance but showed a marginal trend (RR = 1.37, *p* = 0.057).

### Disease severity and laboratory indicators

**Figure 3:**
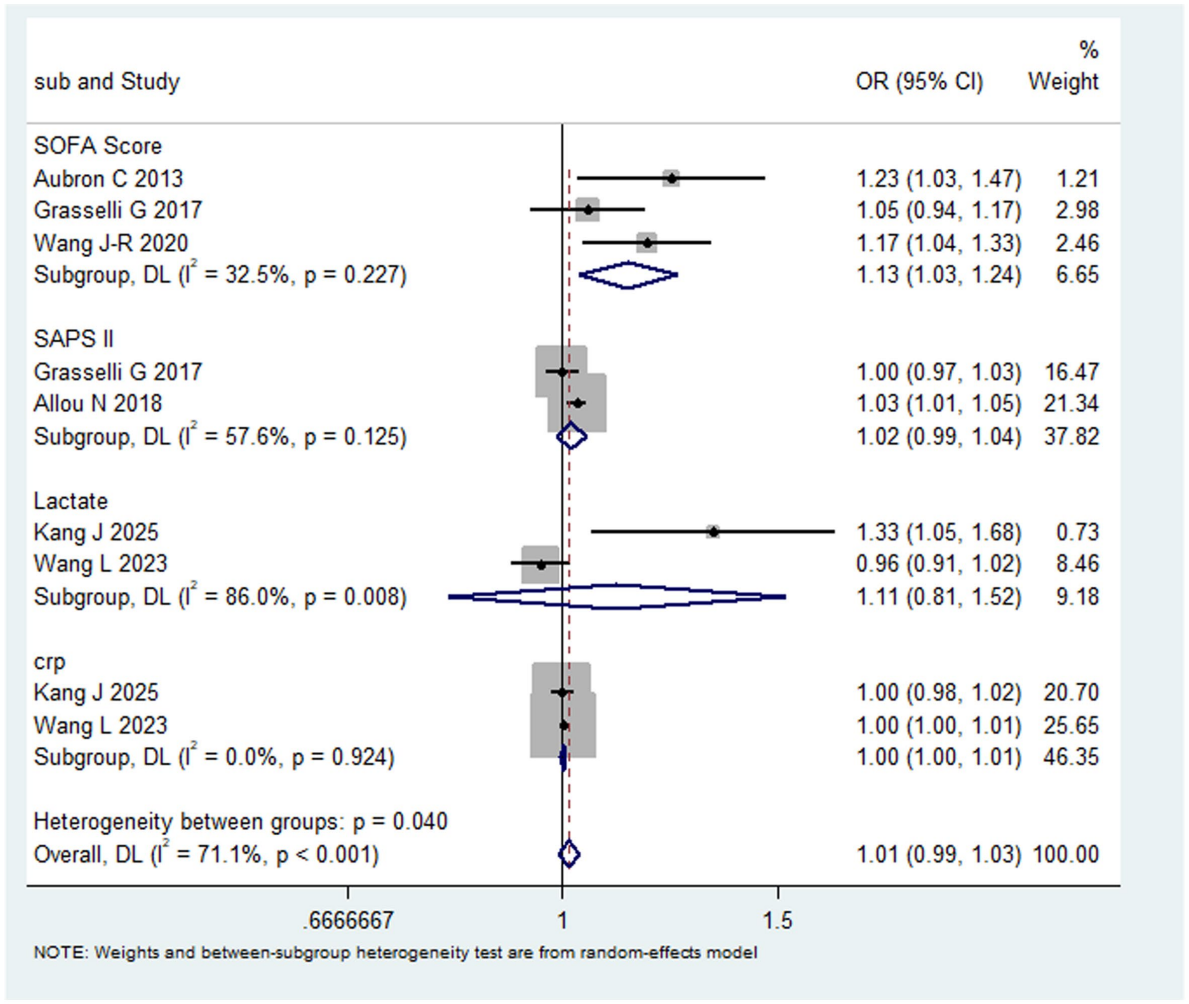
Forest plot summary disease severity and laboratory indicators.

A total of five studies were included for SOFA score, with three providing ORs for analysis (Figure 3). Each one-point increase in SOFA score was associated with a 13% increase in infection risk (pooled OR = 1.13, 95% CI 1.03–1.24, *p* = 0.011) with low heterogeneity (I^2^ = 32.5%). One study reported a multivariable-adjusted HR of 1.04 (95% CI 1.00–1.08, *p* < 0.05), confirming its role as an independent risk factor, while another reported a non-significant RR of 1.00 (95% CI 0.96–1.05, *p* = 0.696). For the SAPS II score, three studies were included with two providing ORs. The trend for increased risk did not reach statistical significance (OR = 1.02, 95% CI 0.99–1.04, *p* = 0.106). However, one study using Cox regression analysis identified SAPS II as an independent risk factor for infection (HR = 1.021, 95% CI 1.01–1.04, *p* < 0.05). The discrepancy in statistical significance between the pooled OR and the adjusted HR may be due to the OR not accounting for the time-to-event nature of infection. Trends for blood lactate, C-reactive protein (CRP), and body mass index (BMI) did not reach statistical significance. Homogeneity for lactate and BMI was extremely poor, suggesting that the direction of effect may be inconsistent across the included studies.

### ECMO and treatment related factors

**Figure 4:**
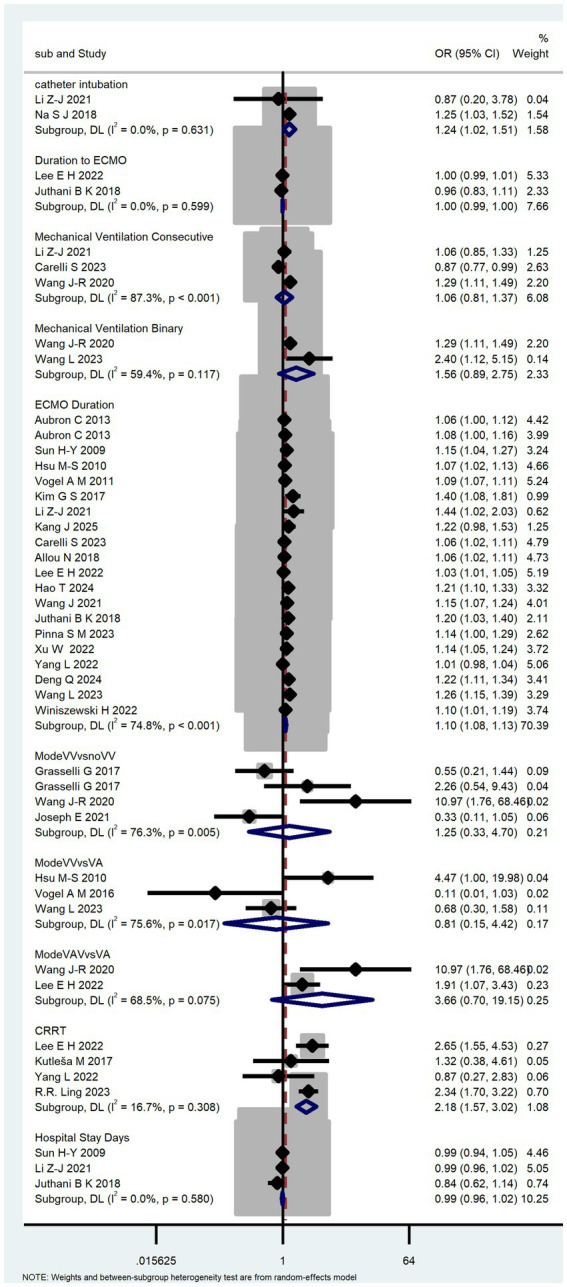
Pooled forest plot of ECMO and treatment-related factors. (1) Virus: Admission due to viral infection. (2) Comorbidities: 1. Mechanical complications included oxygenator failure; pump malfunction; heater dysfunction; clot of oxygenator, hemofilter, and other sites; connector crack; and so forth. 2. Pulmonary complications included pneumothorax and pulmonary hemorrhage. 3. Metabolic complications included blood glucose level <40 mg/dL or>240 mg/dL, serum pH < 7.2 or>7.6, or serum bilirubin level>15 mg/dL. (3) Infection: resent prior to the initiation of extracorporeal membrane oxygenation (ECMO), but with a causative pathogen that differs from the target infection. (4) Catheter intubation: Various types of drainage tubes or invasive monitoring catheters that are left in place in the body, excluding central venous catheters.

Twenty studies were included, with 18 providing ORs for the analysis of ECMO duration (Figure 4). ECMO duration was one of the most robust predictors of infection; the pooled risk estimate per additional day was 1.10 (95% CI 1.07–1.14, *p* < 0.001). Although heterogeneity was high (I^2^ = 76.9%), the direction of effect was highly consistent across the 18 analyses. Furthermore, sensitivity analyses using cut-offs of 72 h, 144 h, 10 days, or 250 h consistently demonstrated an increasing risk of infection with longer ECMO duration. Five studies examined mechanical ventilation as a variable. When mechanical ventilation duration was treated as a continuous variable(For each additional day) in two studies, the pooled effect did not reach significance (OR = 1.56, 95% CI 0.89–2.75). When analyzed as a binary variable (use of mechanical ventilation), individual studies showed significant associations with increased risk (Wang J-R 2020: OR = 1.29, 95% CI 1.11–1.49; Wang L 2023: OR = 2.40, 95% CI 1.12–5.15), but the pooled effect was not statistically significant (OR = 1.56, 95% CI 0.89–2.75), with moderate heterogeneity (I^2^ = 59.4%, *p* = 0.117). This contradictory result may be attributed to between-study effect differences and imprecise estimates from small samples. Notably, one multivariable regression study showed that mechanical ventilation for more than 3 days significantly increased infection risk compared to 3 days or less (OR = 7.33, 95% CI 2.86–20.3). For renal replacement therapy (CRRT), five studies were included, with four providing ORs. CRRT was a clear risk factor, with a pooled OR of 2.18 (95% CI 1.57–3.02, *p* < 0.001) and low heterogeneity (I^2^ = 16.7%). Another study reported a non-significant HR of 0.9 (95% CI 0.42–1.92, *p* = 0.696). Catheter intubation was associated with an increased risk, with a pooled OR of 1.24 (95% CI 1.02–1.51, *p* = 0.032) and I^2^ = 0%. For ECMO mode, seven studies were included. A pooled HR from two studies demonstrated that VV-ECMO, compared with VA-ECMO, significantly reduced the risk of nosocomial infection (HR = 0.45, 95% CI 0.21–0.93, *p* = 0.031) with no heterogeneity (I^2^ = 0%). In contrast, a pooled OR from five studies did not show a significant difference in infection risk between the two modes (OR = 1.61, 95% CI 0.45–5.81, *p* = 0.464), and heterogeneity was extremely high (I^2^ = 73.9%). For the comparison between VAV-ECMO and VA-ECMO, only two studies reported relevant data. The pooled result suggested a possible trend toward a higher infection risk with the VAV mode, but this did not reach statistical significance (OR = 3.66, 95% CI 0.70–19.15, *p* = 0.126).

### Subgroup analysis

To explore potential sources of heterogeneity in the pooled effect estimates, we performed pre-specified subgroup analyses for factors with four or more studies, stratified by infection type (NSI vs. BSI) and study period (before 2020 vs. 2020 and after). Supplementary Figure S1 summarizes the key statistics and core findings of these analyses. Subgroup analyses revealed significant effect modification. The risk associated with ECMO duration and ECMO mode varied by infection type: the effect of ECMO duration on NSI, such as respiratory infections, was far stronger than its effect on BSI. Meanwhile, the VV mode displayed extremely high risk ratios for specific infection types (VAP/BSI) compared to VA mode, albeit with low precision of evidence. The risk associated with immunosuppression demonstrated a striking period-dependent difference: the pooled OR from studies conducted in 2020 and after was more than three times higher than that from earlier studies, with no statistical heterogeneity within either subgroup. This strongly suggests that the COVID-19 pandemic and the associated changes in immunomodulatory treatment practices have substantially potentiated immunosuppression as a risk factor for infection. In contrast, age showed no significant association in any subgroup, further confirming that it is not a risk marker for infection in the ECMO population. For CRRT, the number of studies within subgroups was too small for the stratified results to be sufficiently convincing, although its overall effect was robust. In summary, infection type and study period partially explain the heterogeneity in risk factors for ECMO-related infection. However, for certain factors (e.g., ECMO mode and ECMO duration after 2020), substantial heterogeneity remains unexplained by these two variables, suggesting that deeper confounders such as patient baseline characteristics, cannulation strategies, and center-specific practices warrant further investigation.

### Sensitivity analysis

Given the small number of studies for most factors, no additional sensitivity analysis was performed for those with three or fewer studies owing to limited samples and low heterogeneity (I^2^ ≤ 50%). Supplementary Figure S2 presents the leave-one-out sensitivity analysis. The analysis showed that, except for the mechanical comorbidities group, where the 95% CI crossed the null after the removal of studies such as Li Z-J 2021 (0.72–1.55), the pooled effect estimates for all other indicators—including sex, length of hospital stay, viral infection, infection, CRRT, SOFA, ECMO mode, ECMO duration, immunosuppression, age, and mechanical ventilation—did not change in direction or significance after the removal of any single study, indicating that the overall results of this meta-analysis are robust.

### Publication bias assessment

Because ECMO duration was the only factor with data from more than 10 studies, a formal publication bias test was performed for this factor. Both Egger’s test (bias = 2.588, t = 5.72) and Begg’s test (z = 3.27, continuity corrected) were highly significant (*p* ≤ 0.001), and the funnel plot displayed an asymmetric distribution, indicating significant publication bias (Supplementary Figure S3). The pattern of bias, where smaller studies tended to report larger effect sizes (as confirmed by the intercept of Egger’s test), suggests that the strength of the association between ECMO duration and infection risk may have been overestimated to some extent.

### Descriptive analysis of other risk factors

Several factors could not be pooled because of incompatible effect measures or data errors. For ARDS, bacterial (RR 0.64, 0.40–0.86) and other (RR 0.59, 0.42–0.98) etiologies were associated with lower NSI risk compared to viral ARDS (27). For blood transfusion, one study reported a non-significant association (OR 1.01, 1.00–1.02 per unit) (28) and the other was excluded due to data error (38). For interventions, MSD vs. standard care lowered NSI risk (RR 0.4, 0.24–0.64) (27) and CHG/IPA use lowered BSI risk (OR 0.16, 0.03–0.76) (39), but different effect measures preclude meta-analysis.

Single-study factors associated with increased infection risk were: transthoracic cannulation (BSI: OR 7.11, 1.02–49.45) (33); pH 7.29–7.36 (NSI: OR 1.57, 1.04–2.38) (24); APACHE II ≥ 24 (MDR infection: HR 6.44, 1.38–30.09) (20); MDR Gram-negative colonization (NSI: OR 12.9, 3.41–49.1) (2); CPR per minute (NSI: OR 1.04, 1.01–1.06) (19); surgical indication (HR 2.5, 1.1–5.4; Joseph E 2021); creatinine per 1 mg/dL (OR 2.18, 1.07–4.45) (8); autoimmune diseases (OR 7.00, 1.54–31.77) (7); AKI (BSI: OR 1.62, 1.62–4.24) (15); vasopressor per day (BSI: OR 1.06, 1.06–1.13) (15); oxygenator replacement (BSI: OR 5.13, 1.32–20.00); (39); ECMO system PLS vs. EBS (BSI: OR 1.66, 1.66–3.18) (22); Black vs. White race (NSI: OR 22.36, 2.03–246.63) (17); age per year (NSI: RR 1.17, 1.03–1.33) (27); massive hemorrhage (BSI: OR 3.38, 1.06–10.87) (21); Murray score per point (BSI: OR 6.29, 1.71–23.10) (38).

Protective factors were: antibiotic prophylaxis covering Pseudomonas (NSI: HR 0.51, 0.28–0.94) (20); PEEP 14–16 vs. ≤12 cmH₂O (NSI: OR 0.62, 0.42–0.92) (24); absence of SIRS (HR 0.34, 0.15–0.75) (12); higher bicarbonate levels (OR 0.51, 0.28–0.92) (24). Non-significant associations were found for CVC duration per day (HR 1.01, 1.00–1.02) and platelet count per 1,000/mm^3^ (HR 0.99, 0.98–1.00) (25). All these findings should be interpreted cautiously, as they come from limited (often single-study) evidence.

## Discussion

This meta-analysis of 34 observational studies encompassing approximately 8,901 adult ECMO patients confirms that the risk of NSI is multifactorial, being driven by illness severity (elevated SOFA score), specific comorbidities (type 2 diabetes mellitus, pre-existing microbial colonization), and the duration and complexity of invasive support (ECMO duration, CRRT, mechanical ventilation, central venous catheter placement). Our analysis is the first to establish inter-hospital transport and CVC placement as statistically significant independent risk factors, thereby expanding the recognized risk spectrum.

These findings are highly consistent with those of previous meta-analyses. The strong associations between prolonged ECMO duration, CRRT, mechanical ventilation, and higher SOFA score and NSI risk are in line with the work of Li et al., Lv et al., and Ait Hssain et al. (40–42); notably, the pooled OR for CRRT (2.00) closely approximates the 2.10 reported by Ait Hssain et al. Diabetes mellitus as an independent risk factor is also consistent with the evidence linking hyperglycaemia to impaired immune function (43, 57). Most importantly, this meta-analysis is the first to statistically confirm inter-hospital transport as independent risk factors, thus expanding the catalog of modifiable risks that should be incorporated into quality-improvement initiatives.

Three recently published high-quality studies provide direct context for our results. The multicenter RANGER 2.0 study demonstrated that co-colonization with multiple fungal species independently predicts mortality in patients undergoing V-V ECMO, reinforcing our conclusion that pre-existing microbial colonization (pooled OR 2.95) constitutes a clinically actionable predictor (44). The companion RANGER analysis further revealed that patients with pre-ECMO MDR Gram-negative bacterial colonization or infection experience substantially worse survival than those who acquire MDR during the ECMO run (45). These findings underscore that the timing of colonization is critical and support the implementation of active surveillance cultures upon ICU admission. Our subgroup analysis shows that the risk associated with immunosuppression became markedly stronger in studies conducted from 2020 onward (pooled OR more than threefold higher), likely reflecting the profound impact of the COVID-19 pandemic, during which widespread use of corticosteroids and other immunomodulatory agents altered host susceptibility and expanded the microbiological reservoir. A recent meta-analysis of prone positioning during V-V ECMO revealed that, although proning improves survival, it is also associated with longer ECMO runs (46). This highlights a central clinical tension: strategies that necessarily prolong extracorporeal support may inadvertently increase pathogen exposure, a dynamic consistent with the strong, duration-dependent infection risk we identified.

### Subgroup analyses: rationale and clinical significance

All subgroup analyses reported in this study were pre-specified on the basis of clinical rationale, rather than being driven by post-hoc significance seeking. Stratification by infection type—the most clinically informative analysis—revealed clear effect modification: the association between ECMO duration and NSI was substantially stronger for respiratory and surgical-site infections than for bloodstream infections, suggesting that prolonged instrumentation of the airway and surgical wounds plays a predominant mechanistic role and directly addressing the clinical need to determine which infection type drives overall risk. This finding implies that prevention bundles should be tailored according to the dominant infection type in each unit (e.g., prioritizing VAP prevention in centers with long ECMO runs) (60). Stratification by study period (pre-2020 vs. 2020 and thereafter) was designed to capture the epidemiological shift brought about by the COVID-19 pandemic and revealed a pronounced increase in immunosuppression-related risk, confirming that era-specific effects must be considered when extrapolating historical data to contemporary practice.

### Clinical implications: a stratified prevention framework

The multifactorial nature of NSI risk demands a stratified prevention strategy that distinguishes among modifiable, avoidable, and non-modifiable factors. Actively reduce modifiable risk factors: The core principle remains the systematic minimization of the duration of all invasive devices. ECMO, mechanical ventilation, CVCs, and CRRT should each be targeted through rigorous daily assessment for weaning readiness and prompt removal when no longer indicated (58, 59). Adherence to evidence-based catheter insertion and maintenance bundles, daily chlorhexidine gluconate bathing for high-risk patients (49–54).

Avoid high-risk interventions and practices (50, 51): When peripheral access can meet therapeutic needs, unnecessary CVC placement should be avoided, as CVCs independently increase infection risk. Routine prophylactic antibiotic administration is not recommended (55, 56), as current evidence does not support it and there are concerns regarding selection for MDR organisms. Surgical cannulation strategies that may elevate infection risk should be reserved for cases in which percutaneous access is contraindicated (51).

Proactively prevent infection in patients with non-modifiable risks: For factors that cannot be readily modified—such as age, underlying disease severity (SOFA), pre-existing immunosuppression, or the need for inter-hospital transport—intensified surveillance and pre-emptive measures are essential. Our results, together with the recent RANGER data (44, 45), strongly support the implementation of active microbiological surveillance for MDR and fungal colonization, particularly in patients transferred from external facilities or those with prolonged ICU stays, because colonization more than doubles infection risk. In settings with high MDR prevalence, selective oropharyngeal or digestive decontamination may be considered as a unit-level strategy under the guidance of antimicrobial stewardship policies (55, 56, 61, 62). Hemodynamic management should aim to avoid prolonged vasopressor dependence, which is itself associated with infection. For patients who must be transported, strict adherence to sterile technique during transfer and handover is essential. This three-tiered framework, integrating active risk reduction, avoidance of unnecessary exposure, and proactive mitigation of immutable risks, is more likely to achieve a meaningful reduction in NSI incidence than any single intervention alone.

### Interpretation of heterogeneity

The substantial heterogeneity observed for multiple risk factors arises from both methodological and clinical sources, and distinguishing between the two has important implications. For ECMO duration, meta-regression identified the small-study effect as the dominant source of variance (explaining 89.09% of the between-study variance), which is consistent with the significant publication bias detected. Therefore, while the pooled OR for ECMO duration may be inflated, the consistency in the direction of effect across all studies supports a genuine biological association. In contrast, the high heterogeneity for immunosuppression and microbial colonization is more likely attributable to genuine clinical diversity. A key source of this diversity is the lack of standardized infection definitions. The ELSO registry captures only culture data without uniform clinical criteria, and individual studies employ varying diagnostic thresholds for nosocomial events. This definitional variability, interacting with differences in local microbiological epidemiology and center-specific infection prevention practices, means that the pooled estimate represents only an approximate average across fundamentally dissimilar settings (47). Advancing consensus definitions for ECMO-related infections must therefore be a priority for improving the comparability of future meta-analyses (48).

### Methodological considerations

The use of different effect measures HR, OR can yield divergent results. This is exemplified by the immunosuppression analysis, in which the OR subgroup and the HR subgroup showed opposite directions of effect. Accordingly, we performed separate meta-analyses by effect measure type wherever feasible. With respect to model selection, although a random-effects model was adopted for analyses with I^2^ ≥ 50%, we recognize that the conceptual heterogeneity arising from differences in infection definitions, ECMO populations, and clinical settings provides a strong rationale for using random-effects models as the default throughout (47, 48).

### Limitations

This meta-analysis has several limitations. Despite the consistent inclusion of multivariable-adjusted estimates, retrospective, single-center studies remain predominant, which may introduce selection bias and information bias. Because of the lack of standardized infection definitions and the heterogeneity of ECMO populations, substantial residual heterogeneity persists for several factors. The limited number of studies within each infection-type subgroup restricted the power to detect subtle effect modifications. Moreover, the included studies span more than two decades, and ECMO technology has advanced rapidly, particularly after 2020; this may introduce temporal bias due to developments in extracorporeal membrane oxygenation technology and critical care practices. Prospective, multicenter studies using consensus infection definitions are essential to validate these findings.

## Conclusion

Nosocomial infection in ECMO patients results from a complex interplay of patient baseline characteristics, illness severity, and the intensity and duration of invasive support. It is important to identify high-risk patient subgroups exhibiting these features and to explore newly identified risk associations. A stratified prevention approach—minimizing modifiable risks, avoiding high-risk practices, and providing proactive protective measures for patients with non-modifiable vulnerabilities—should be implemented. However, given the inherent limitations of the available evidence, which is derived primarily from retrospective studies with significant heterogeneity, prospective research is anticipated to further explore and validate these findings.

## Data Availability

The original contributions presented in the study are included in the article/Supplementary material, further inquiries can be directed to the corresponding author.

## Glossary

ARDS: Acute Respiratory Distress Syndrome
BMI: Body Mass Index
BSI: Bloodstream Infectio
CHG: Chlorhexidine Gluconate
CI: Confidence Interval
CPR: Cardiopulmonary Resuscitation
CRRT: Continuous Renal Replacement Therapy
CSI: Cannulation Site Infection
CVC: Central Venous Catheter
ECMO: Extracorporeal Membrane Oxygenation
HR: Hazard Ratio
ICU: Intensive Care Unit
MDR: Multidrug-Resistant
MSD: Multi-Site Decontamination
NSI: Nosocomial Infection
NOS: Newcastle-Ottawa Scale
OR: Odds Ratio
PRISMA: Preferred Reporting Items for Systematic Reviews and Meta-Analyses
RCT: Randomized Controlled Trial
RR: Risk Ratio
SAPS II: Simplified Acute Physiology Score II
SDD: Selective Digestive Decontamination
SOFA: Sequential Organ Failure Assessment
SOD: Selective Oropharyngeal Decontamination
SSI: Surgical Site Infection
T2DM: Type 2 Diabetes Mellitus
UTI: Urinary Tract Infection
VA-ECMO: Veno-Arterial Extracorporeal Membrane Oxygenation
VAP: Ventilator-Associated Pneumonia
VV-ECMO: Veno-Venous Extracorporeal Membrane Oxygenation
IPA: Invasive Pulmonary Aspergillosis
