# Supplementary material for: Risk factors for nosocomial infections in ECMO patients: a systematic review and meta-analysis
Source: Front Public Health. 2026 Jun 11;14:1820017. doi: 10.3389/fpubh.2026.1820017 (PMC13294189; doi:10.3389/fpubh.2026.1820017)

Figure S1A. Forest plot: subgroup by age (cut-off at 2020).


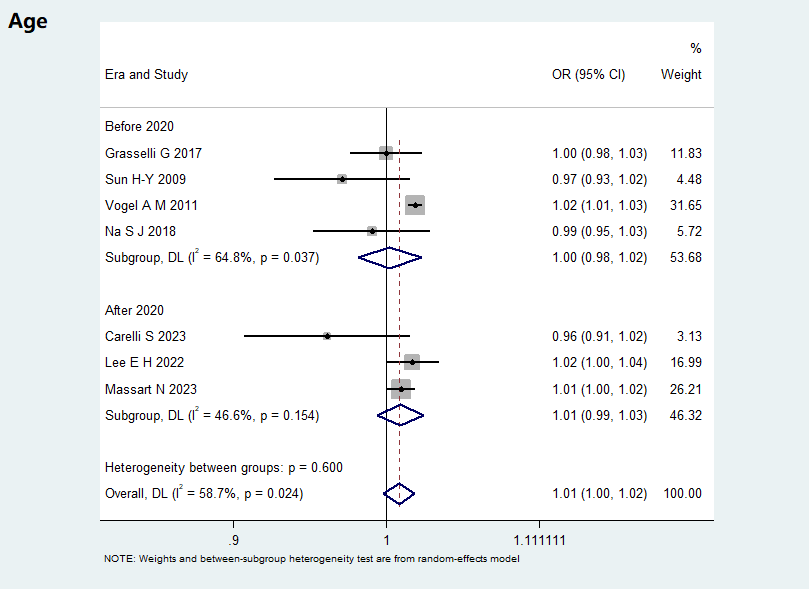


Figure S1B. Forest plot: subgroup by age (Infection type).


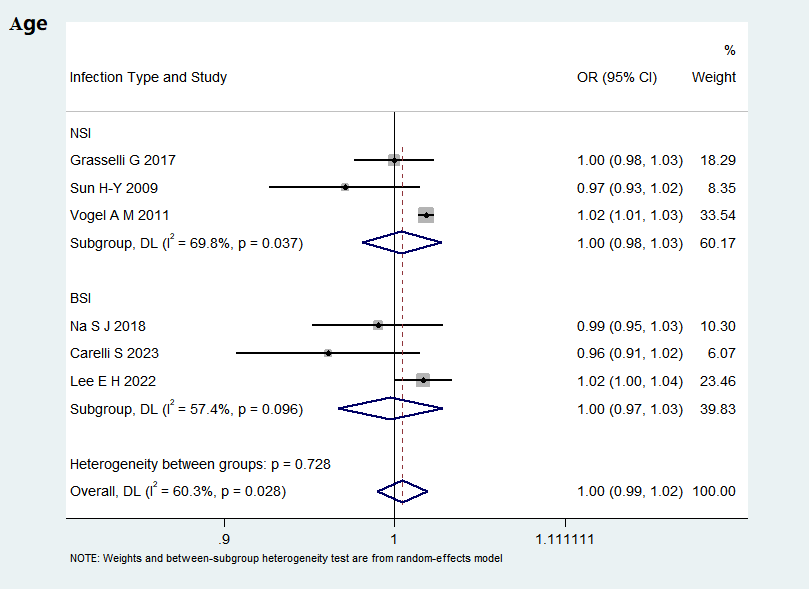


Figure S1C. Forest plot: subgroup by CRRT (Infection type).


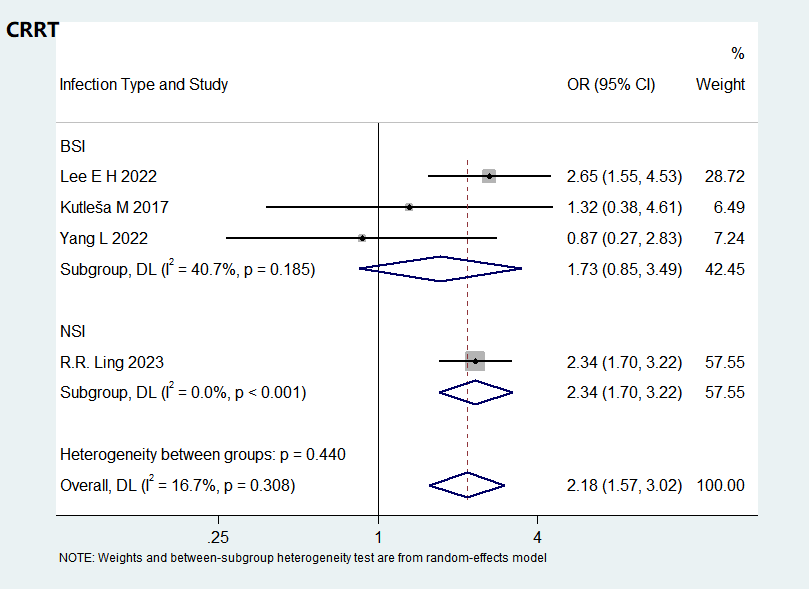


Figure S1D. Forest plot: subgroup by ECMO Duration (Infection type).


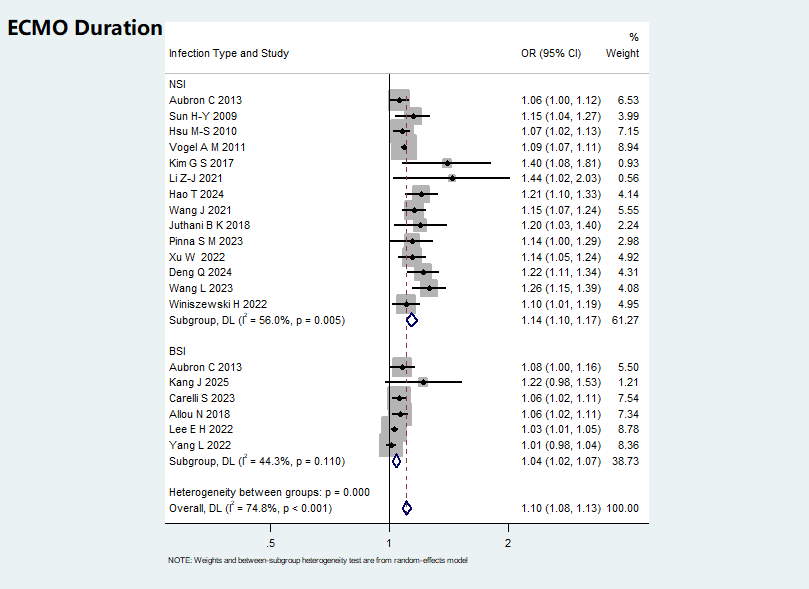


Figure S1E. Forest plot: subgroup by ECMO Duration (cut-off at 2020).


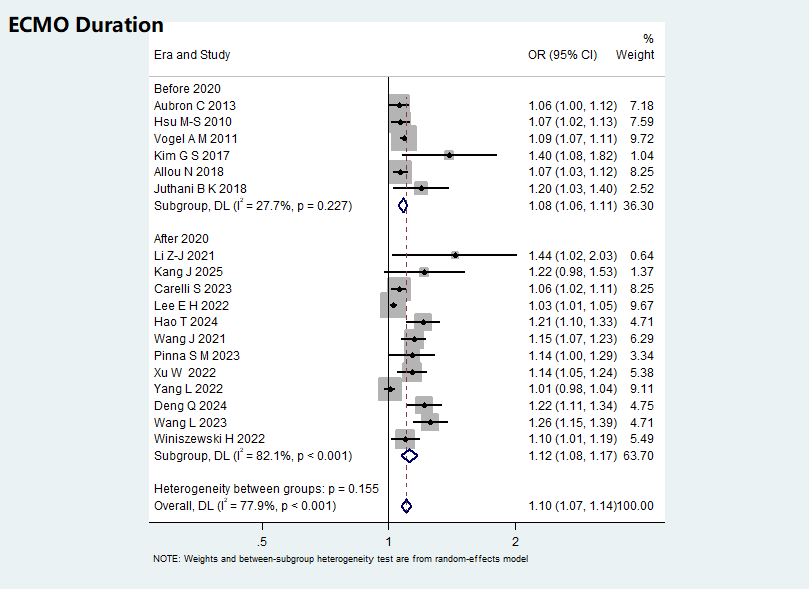


Figure S1F. Forest plot: subgroup by immunosuppression (cut-off at 2020).


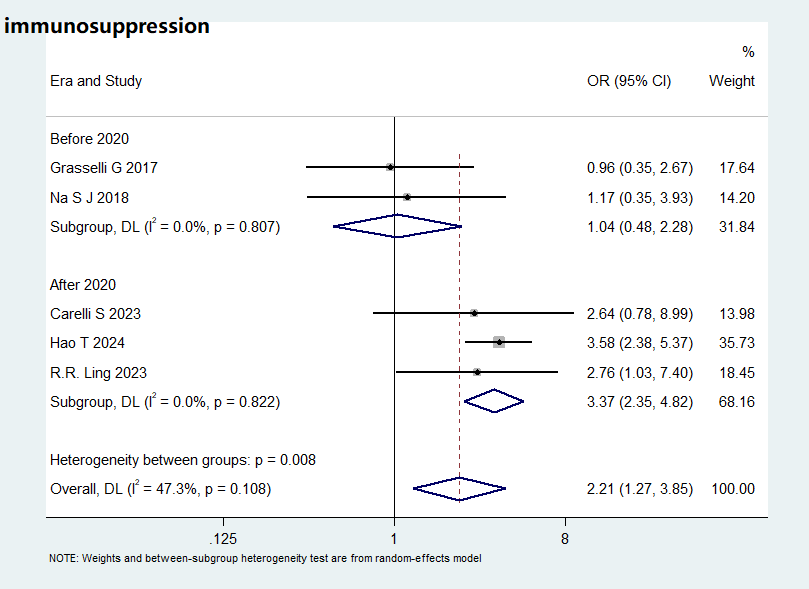


Figure S1G. Forest plot: subgroup by immunosuppression (Infection type).


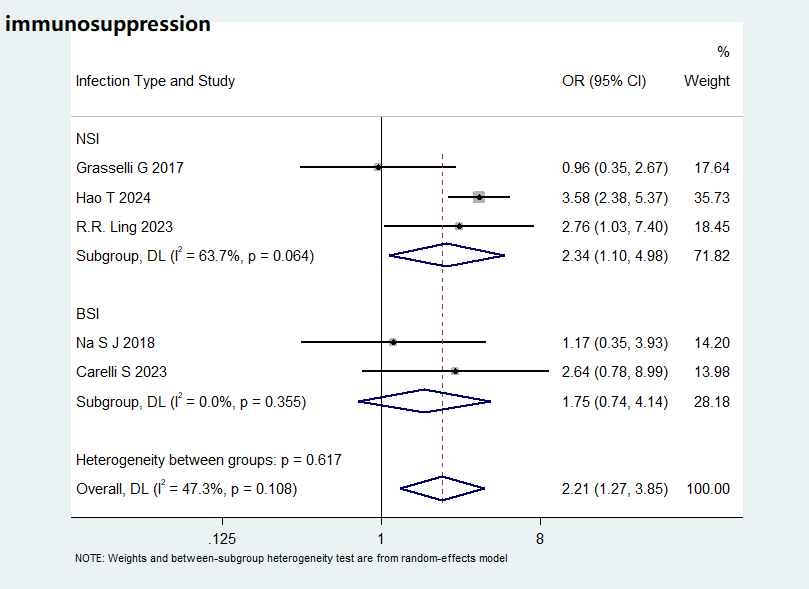


Figure S1H. Forest plot: subgroup by Mode (Infection type).


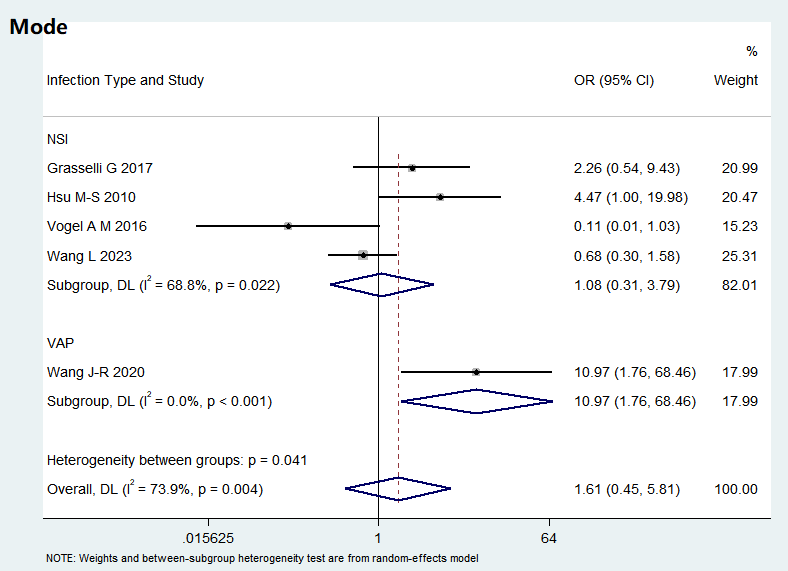


Figure S1I. Forest plot: subgroup by Mode (cut-off at 2020).


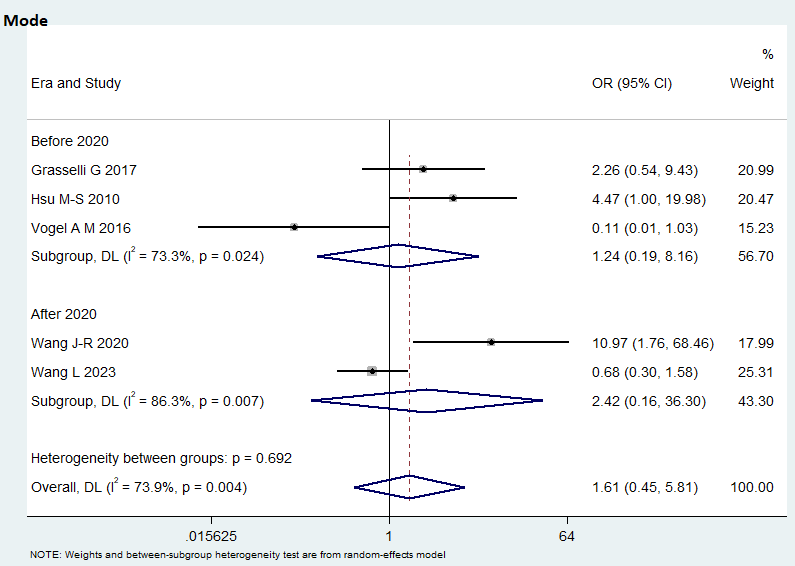

Supplement: Supplementary file 1 [file Supplementary_file_1.docx]
