# Supplementary material for: Risk factors for nosocomial infections in ECMO patients: a systematic review and meta-analysis
Source: Front Public Health. 2026 Jun 11;14:1820017. doi: 10.3389/fpubh.2026.1820017 (PMC13294189; doi:10.3389/fpubh.2026.1820017)

Figure S2A.Sensitivity analysis of Immunosuppression.

Figure S2B.Sensitivity analysis of Mode. Figure S2C.Sensitivity analysis of SOFA. Figure S2E.Sensitivity analysis of Mechanical ventilation. Figure S2F.Sensitivity analysis of Infection.


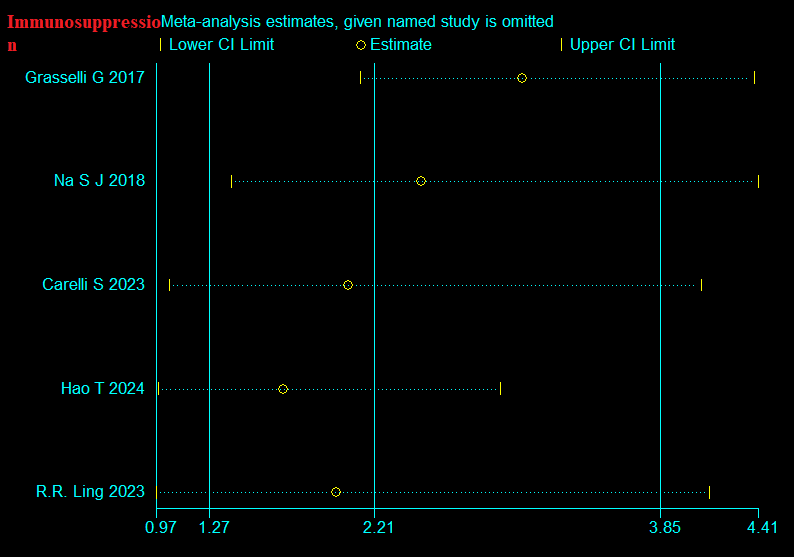

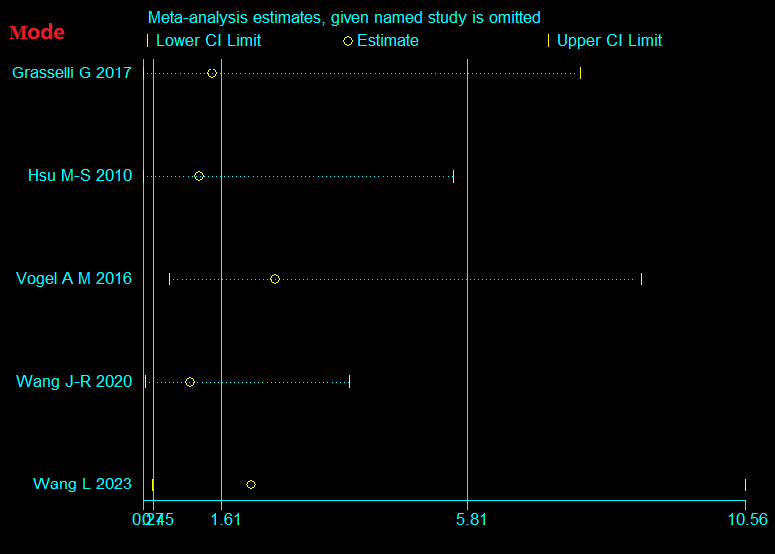

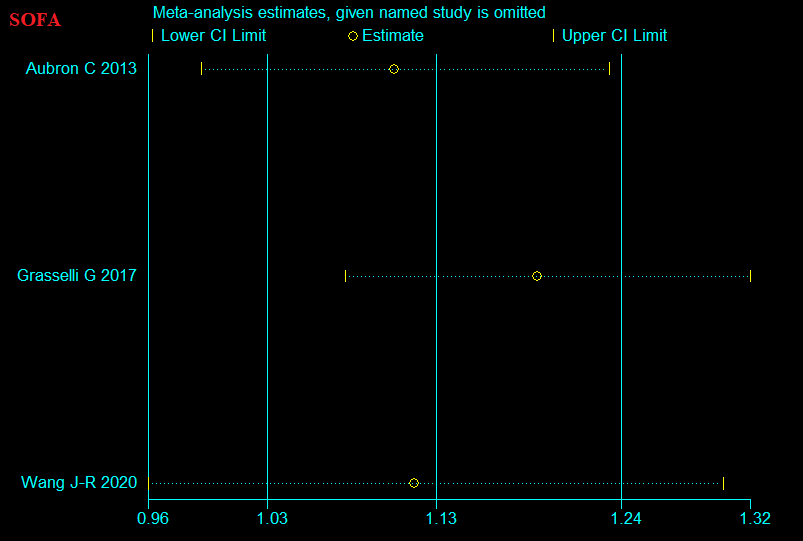

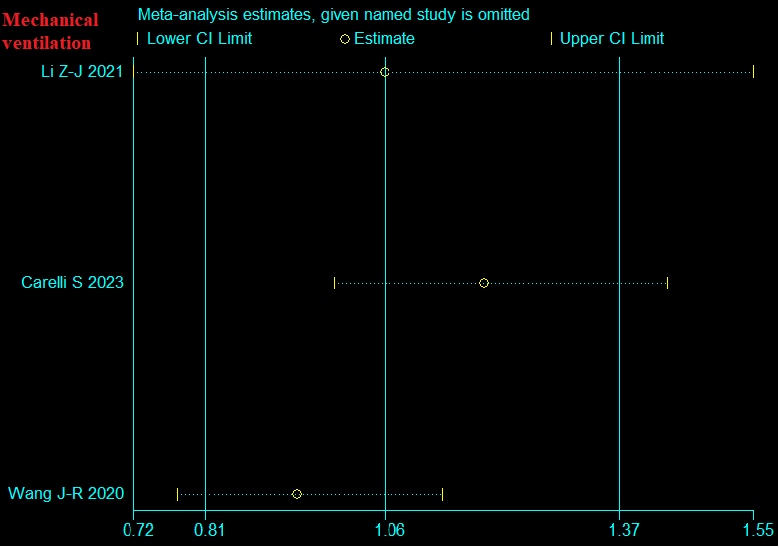

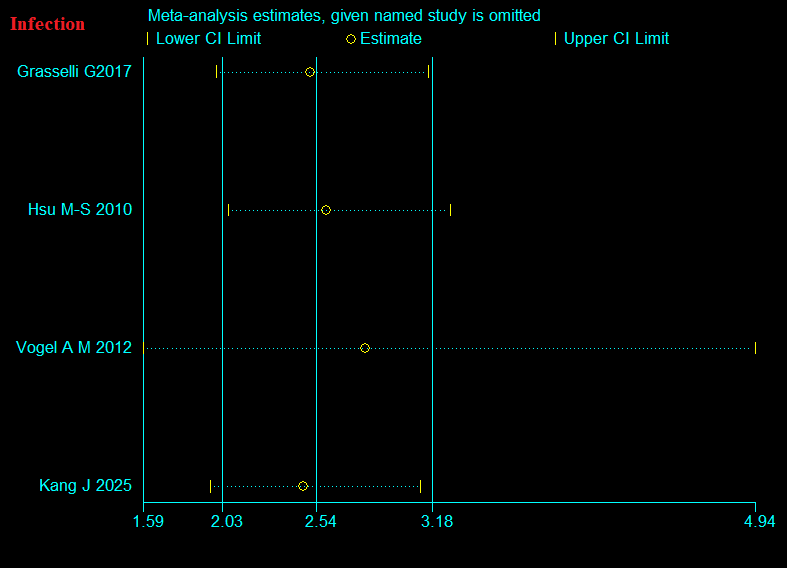


Figure S2G.Sensitivity analysis of Virus. Figure S2H.Sensitivity analysis of Mechanical complications.


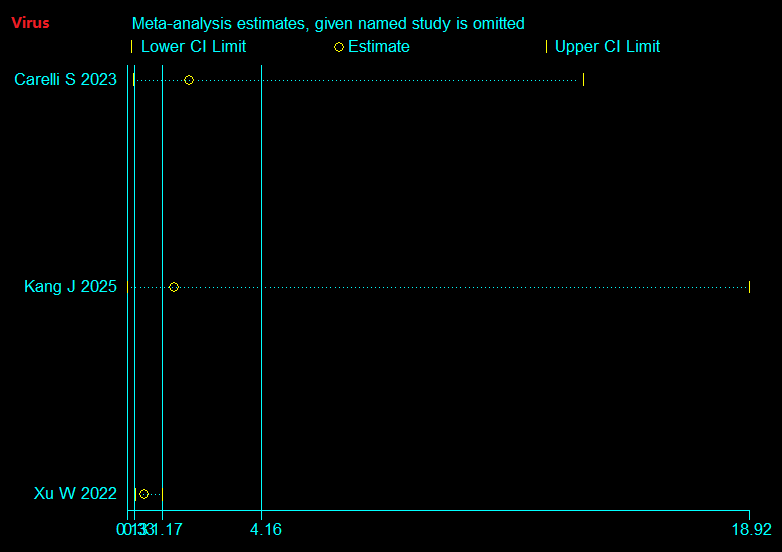

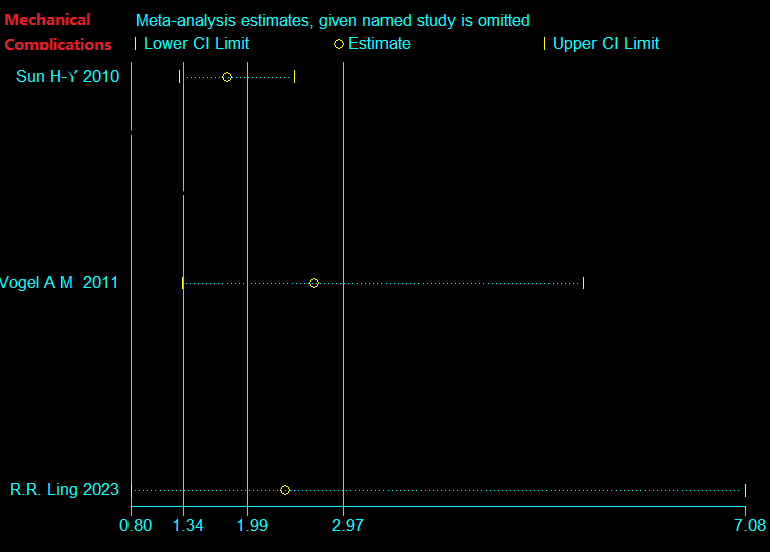


Figure S2I.Sensitivity analysis of Hospital Stay Days.


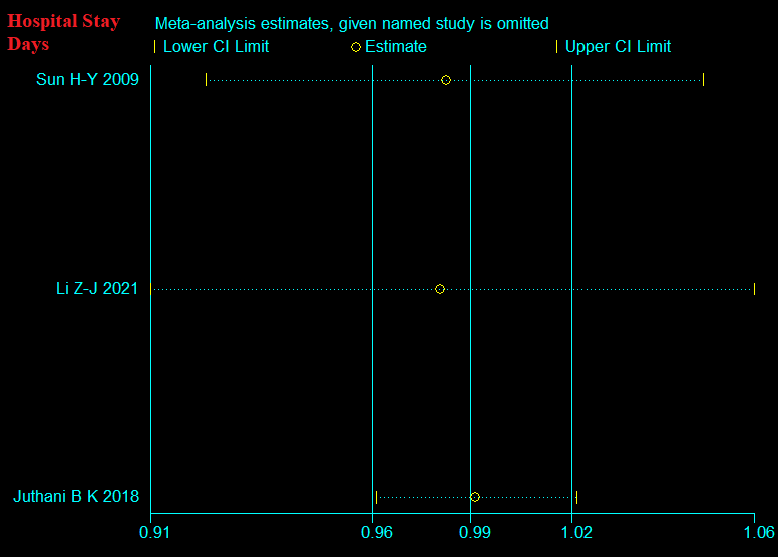


Figure S2J.Sensitivity analysis of Gender.


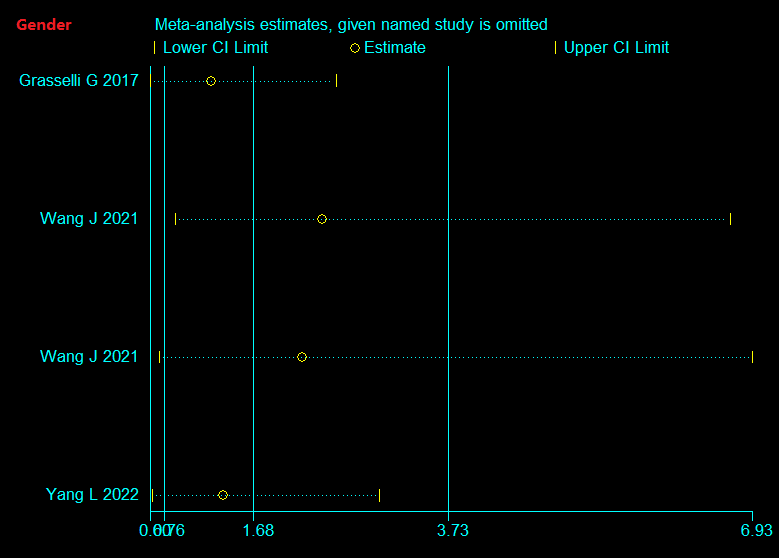


Figure S2K.Sensitivity analysis of Age.


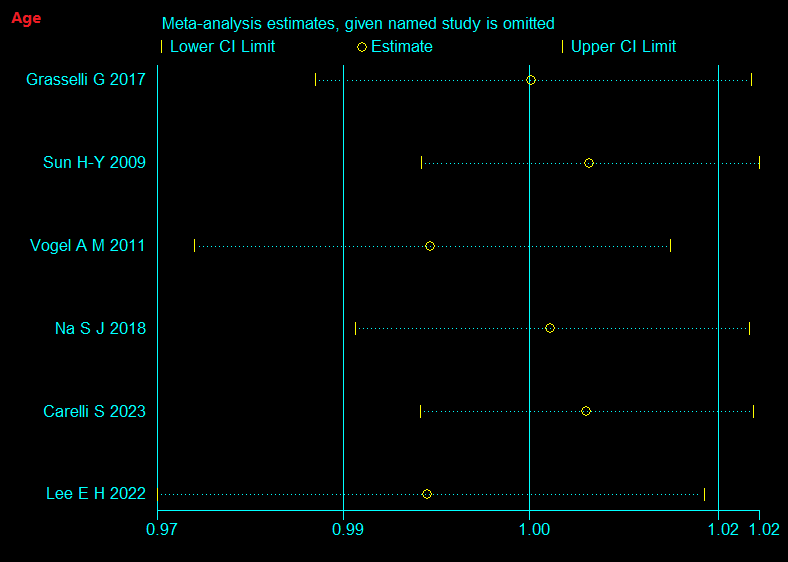


Figure S2L.Sensitivity analysis of Diabetes


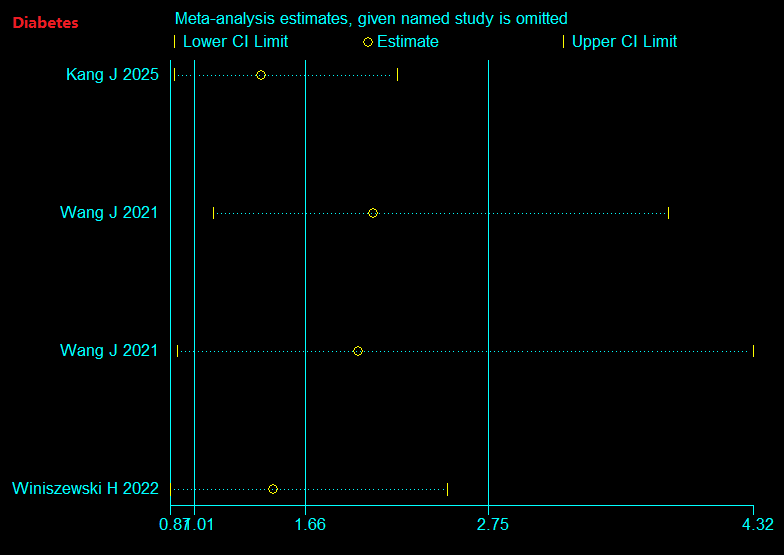


Figure S2M.Sensitivity analysis of Crrt


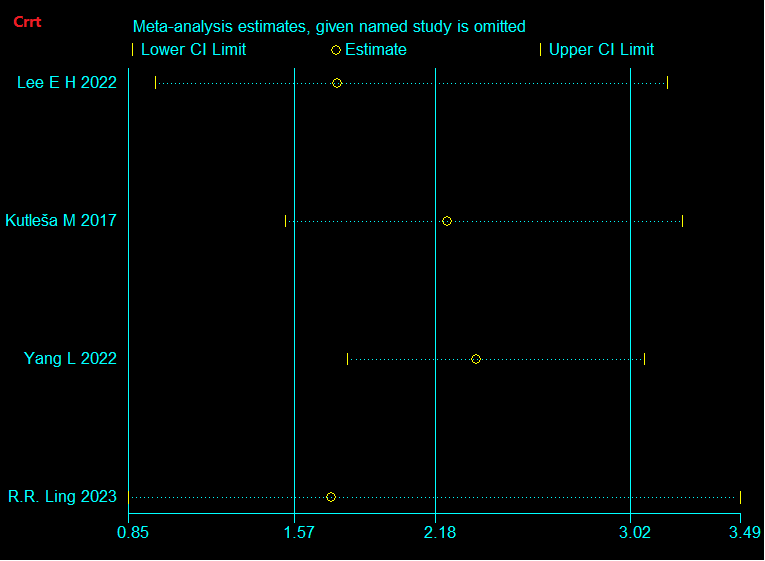


Figure S2N.Sensitivity analysis of ECMO Duration.


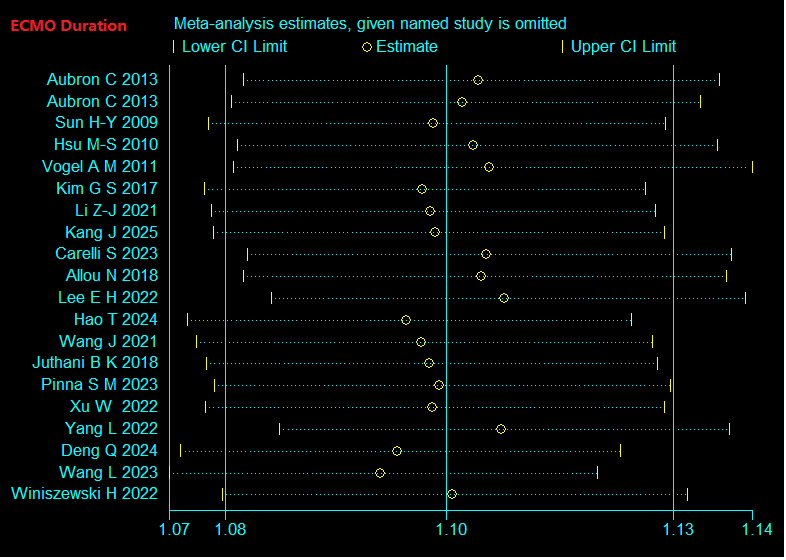

Supplement: Supplementary file 2 [file Supplementary_file_2.docx]
