# Supplementary material for: Risk factors for nosocomial infections in ECMO patients: a systematic review and meta-analysis
Source: Front Public Health. 2026 Jun 11;14:1820017. doi: 10.3389/fpubh.2026.1820017 (PMC13294189; doi:10.3389/fpubh.2026.1820017)

Figure S3.Publication Bias Assessment of ECMO Duration.

### ****A.Trim and Fill Method for Publication Bias Adjustment.****


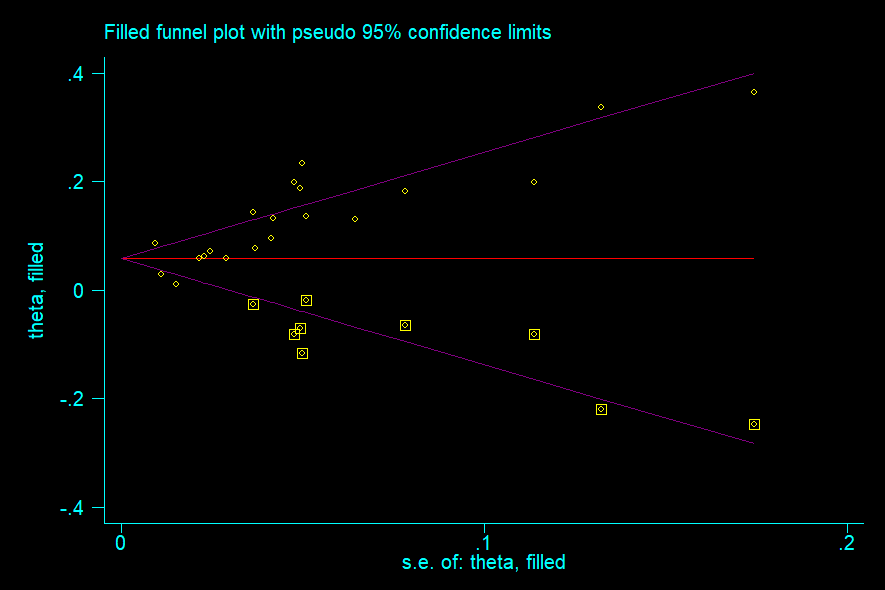


**B.**Funnel Chart for Publication Bias Assessment.


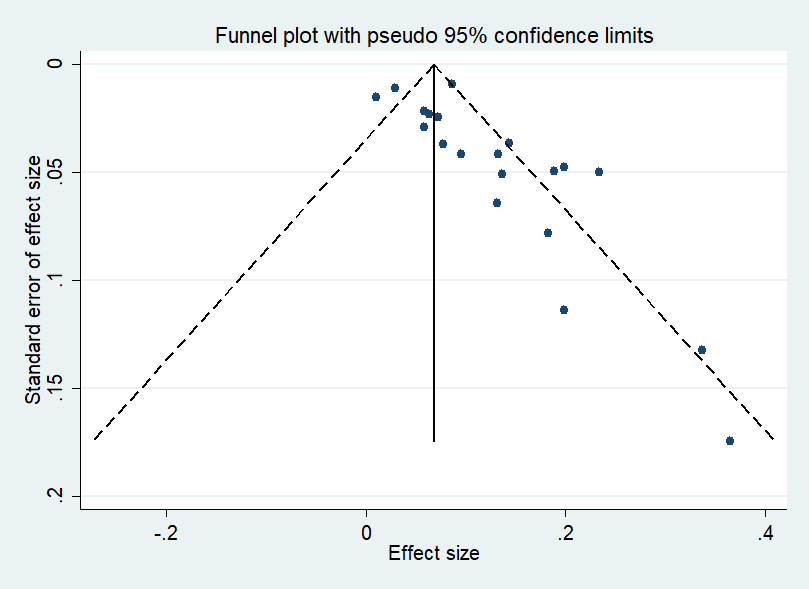
**C.**Egger’s Plot for Publication Bias Assessment.


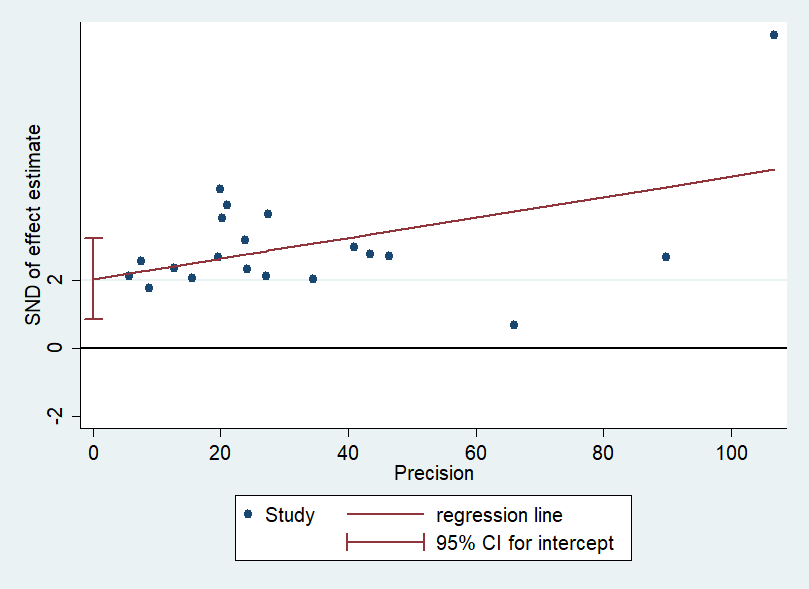


**D.**Sensitivity Analysis for ECMO Duration


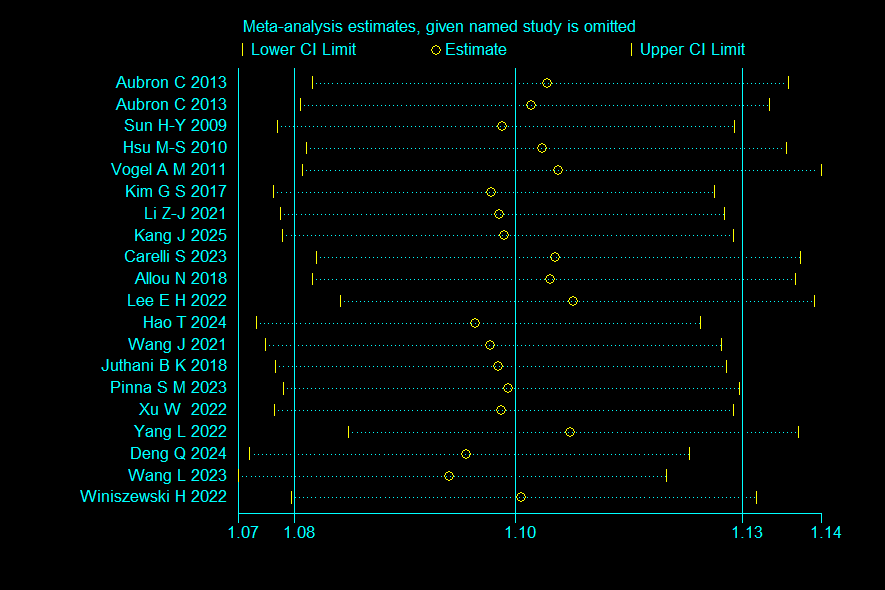

Supplement: Supplementary file 3 [file Supplementary_file_3.doc]
