# Supplementary material for: Risk factors for nosocomial infections in ECMO patients: a systematic review and meta-analysis
Source: Front Public Health. 2026 Jun 11;14:1820017. doi: 10.3389/fpubh.2026.1820017 (PMC13294189; doi:10.3389/fpubh.2026.1820017)
Supplement: Supplementary file 4 [file Table_1.docx]

Table S1.Search strategy

| **Database** | **Search Strategy** | **Results** | **Date** |
| --- | --- | --- | --- |
| **PubMed**​ | ((((Factor, Risk[Title/Abstract]) OR (Risk Factor[Title/Abstract]) OR (Population at Risk[Title/Abstract]) OR (Populations at Risk[Title/Abstract]) OR (Risk Scores[Title/Abstract]) OR (Risk Score[Title/Abstract]) OR (Score, Risk[Title/Abstract]) OR (Risk Factor Scores[Title/Abstract]) OR (Risk Factor Score[Title/Abstract]) OR (Score, Risk Factor[Title/Abstract]) OR (Health Correlates[Title/Abstract]) OR (Correlates, Health[Title/Abstract]) OR (Social Risk Factors[Title/Abstract]) OR (Factor, Social Risk[Title/Abstract]) OR (Factor, Social Risk[Title/Abstract]) OR (Social Risk[Title/Abstract]) OR (Risk Factor, Social[Title/Abstract]) OR (Risk Factors, Social[Title/Abstract]) OR (Social Risk Factor[Title/Abstract]) OR "Risk Factors"[Mesh]) AND ((Extracorporeal Membrane Oxygenation[Mesh]) OR (Extracorporeal Membrane Oxygenations[Title/Abstract]) OR (Membrane Oxygenation, Extracorporeal[Title/Abstract]) OR (ECMO Extracorporeal Membrane Oxygenation[Title/Abstract]) OR (Extracorporeal Life Support[Title/Abstract]) OR (Extracorporeal Life Supports[Title/Abstract]) OR (Life Support, Extracorporeal[Title/Abstract]) OR (ECMO Treatment[Title/Abstract]) OR (ECMO Treatments[Title/Abstract]) OR (Treatment, ECMO[Title/Abstract]) OR (ECLS Treatment[Title/Abstract]) OR (ECLS Treatments[Title/Abstract]) OR (Treatment, ECLS[Title/Abstract]) OR (Oxygenation, Extracorporeal Membrane[Title/Abstract]) OR (Venoarterial ECMO[Title/Abstract]) OR (ECMO, Venoarterial[Title/Abstract]) OR (Venoarterial ECMOs[Title/Abstract]) OR (Venoarterial Extracorporeal Membrane Oxygenation[Title/Abstract]) OR (Venovenous ECMO[Title/Abstract]) OR (ECMO, Venovenous[Title/Abstract]) OR (Venovenous ECMOs[Title/Abstract]) OR (Venovenous Extracorporeal Membrane Oxygenation[Title/Abstract]) OR (ECMO[Title/Abstract])) AND (("Infections"[Mesh]) OR (Infection[Title/Abstract]) OR (Infestation[Title/Abstract]) OR (Infestation and Infection[Title/Abstract]) OR (Infections and Infestations[Title/Abstract]) OR (Infestations and Infections[Title/Abstract]))) | 3,035 | 2025-09-24 |
| **Embase**​ | **#1**​ 'risk factor'/exp OR 'relative risk' OR 'risk factors' OR 'risk factor' **#2**​ 'extracorporeal oxygenation'/exp OR 'ecls (extracorporeal life support)' OR 'ecls therapy' OR 'ecls treatment' OR 'ecmo (extracorporeal membrane oxygenation)' OR 'ecmo support' OR 'ecmo therapy' OR 'ecmo treatment' OR 'extra corporal membrane oxygenation' OR 'extra corporeal life support' OR 'extra corporeal membrane oxygen support' OR 'extra corporeal membrane oxygenation' OR 'extra corporeal membrane oxygenator support' OR 'extra corporeal membrane oxygenator therapy' OR 'extra corporeal membranous oxygenation' OR 'extra corporeal oxygenation' OR 'extra-corporeal membrane oxygenation' OR 'extra-pulmonary oxygen therapy' OR 'extra-pulmonary oxygenation' OR 'extra-pulmonic oxygenation' OR 'extracorporal membrane oxygenation' OR 'extracorporal oxygenation' OR 'extracorporal oxygenization' OR 'extracorporeal circulation membrane oxygen support' OR 'extracorporeal life support' OR 'extracorporeal membrane oxygenation' OR 'extracorporeal membrane oxygen (therapy)' OR 'extracorporeal membrane oxygen support' OR 'extracorporeal membrane oxygen-ation' OR 'extracorporeal membrane oxygenation' OR 'extracorporeal membrane oxygenaton' OR 'extracorporeal membraneous oxygenation' OR 'extracorporeal membranes oxygenation' OR 'extracorporeal membranooxygenation' OR 'extracorporeal membranous oxygen support' OR 'extracorporeal membranous oxygenation' OR 'extracorporeal membranous oxygenator support' OR 'extracorporeal membranooxygenation' OR 'extracorporeal pump oxygenation' OR 'extrapulmonary blood oxygenation' OR 'extrapulmonary membrane oxygenation' OR 'extrapulmonary oxygen therapy' OR 'extrapulmonary oxygenation' OR 'membrane oxygenation, extracorporeal' OR 'oxygenation, extracorporeal' OR 'extracorporeal oxygenation' **#3**​ 'infection'/exp OR 'accidental infection' OR 'acute infection' OR 'autoinfection' OR 'bacterial infections and mycoses' OR 'bacteroid infection' OR 'chain of infection' OR 'infection mechanism' OR 'infection route' OR 'infections' OR 'infectious disease' OR 'route of infection' OR 'infection' **#4**​ #1 AND #2 AND #3 | 3,087 | 2025-09-24 |
| **Cochrane Library**​ | **#1**​ (Venovenous ECMO):ti,ab,kw OR (Venovenous Extracorporeal Membrane Oxygenation):ti,ab,kw OR (ECMO, Venovenous):ti,ab,kw OR (Venovenous ECMOs):ti,ab,kw OR (ECMO, Venoarterial):ti,ab,kw OR (Venoarterial Extracorporeal Membrane Oxygenation):ti,ab,kw OR (Venoarterial ECMOs):ti,ab,kw OR (Venoarterial ECMO):ti,ab,kw OR (Membrane Oxygenation, Extracorporeal):ti,ab,kw OR (ECLS Treatment):ti,ab,kw OR (ECMO Treatment):ti,ab,kw OR (ECMO Treatments):ti,ab,kw OR (Extracorporeal Life Support):ti,ab,kw OR (Treatment, ECMO):ti,ab,kw OR (Life Support, Extracorporeal):ti,ab,kw OR (ECMO Extracorporeal Membrane Oxygenation):ti,ab,kw OR (Extracorporeal Membrane Oxygenations):ti,ab,kw OR (Oxygenation, Extracorporeal Membrane):ti,ab,kw OR (ECLS Treatments):ti,ab,kw OR (Extracorporeal Life Supports):ti,ab,kw OR (Treatment, ECLS):ti,ab,kw **#2**​ MeSH descriptor: [Extracorporeal Membrane Oxygenation] explode all trees **#3**​ MeSH descriptor: [Infections] explode all trees **#4**​ (Infestations and Infections):ti,ab,kw OR (Infection and Infestation):ti,ab,kw OR (Infestation and Infection):ti,ab,kw OR (Infection):ti,ab,kw OR (Infections and Infestations):ti,ab,kw **#5**​ MeSH descriptor: [Risk Factors] explode all trees **#6**​ (Risk Factor):ti,ab,kw OR (Factor, Risk):ti,ab,kw OR (Risk Factor Scores):ti,ab,kw OR (Score, Risk Factor):ti,ab,kw OR (Score, Risk):ti,ab,kw OR (Risk Scores):ti,ab,kw OR (Risk Factor Score):ti,ab,kw OR (Risk Score):ti,ab,kw OR (Risk Factor, Social):ti,ab,kw OR (Factors, Social Risk):ti,ab,kw OR (Social Risk Factor):ti,ab,kw OR (Risk Factors, Social):ti,ab,kw OR (Factor, Social Risk):ti,ab,kw OR (Social Risk Factors):ti,ab,kw OR (Health Correlates):ti,ab,kw OR (Correlates, Health):ti,ab,kw OR (Populations at Risk):ti,ab,kw OR (Population at Risk):ti,ab,kw **#7**​ #1 OR #2 **#8**​ #3 OR #4 **#9**​ #5 OR #6 **#10**​ #7 AND #8 AND #9 | 66 | 2025-09-24 |
| **EBSCO (CINAHL)**​ | **S1**​ (MM "Extracorporeal Membrane Oxygenation" OR ("ECMO" OR "Extracorporeal Membranes Oxygenation" OR "Membrane Oxygenation, Extracorporeal" OR "Oxygenation, Extracorporeal Membrane")) **S2**​ (MM "Infection" OR (TI (infection* OR sepsis OR bacteremia OR "bloodstream infection" OR "surgical site infection" OR "urinary tract infection" OR "respiratory tract infection" OR "nosocomial infection" OR "healthcare-associated infection") OR AB (infection* OR sepsis OR bacteremia OR "bloodstream infection" OR "surgical site infection" OR "urinary tract infection" OR "respiratory tract infection" OR "nosocomial infection" OR "healthcare-associated infection"))) **S3**​ (MM "Risk Factors+" OR MH "Health Status Indicators" OR MH "Risk Control (Iowa NOC)" OR MH "Risk Detection (Iowa NOC)" OR MH "Risk Identification (Iowa NIC)" OR MH "Risk Identification: Childbearing Family (Iowa NIC)" OR MH "Obesity Paradox") **S4**​ S3 AND S2 AND S1 | 3 | 2025-09-24 |
| **Web of Science**​ | **#1**​ TS=(("Extracorporeal Membrane Oxygenation" OR ECMO* OR "Extracorporeal Life Support" OR ECLS*) OR ("Venovenous ECMO" OR "Veno-venous" NEAR/3 (ECMO* OR "Extracorporeal")) OR ("Venoarterial ECMO" OR "Veno-arterial" NEAR/3 (ECMO* OR "Extracorporeal"))) **#2**​ TS=(infection* OR sepsis OR bacteremia OR "bloodstream infection" OR "surgical site infection" OR "urinary tract infection" OR "respiratory tract infection" OR "nosocomial infection" OR "healthcare-associated infection") **#3**​ TS=("risk factor*" OR "determinant*" OR "predictor*" OR "influence factor*" OR "contributing factor*" OR "risk marker*") **#4**​ #1 AND #2 AND #3 | 827 | 2025-09-25 |
