# Supplementary material for: Risk factors for nosocomial infections in ECMO patients: a systematic review and meta-analysis
Source: Front Public Health. 2026 Jun 11;14:1820017. doi: 10.3389/fpubh.2026.1820017 (PMC13294189; doi:10.3389/fpubh.2026.1820017)
Supplement: Supplementary file 5 [file Table_2.docx]

**Table S2:**The number and types of risk factors included in the study.

| **Study** | **Risk factors** |
| --- | --- |
| Aubron C. 2013 | 20，14 |
| Grasselli G2017 | 2,14，16，19，18，12, 7 |
| Bachleda T2015 | 1，12 |
| Hsu M-S2009 | 20，10 |
| Sun H-Y2010 | 1，20，18，5 |
| Vogel A M2011 | 20，19 |
| Schmidt M2012 | 14 |
| Bougle A2018 | 19，2，15 |
| Kim G S2017 | 20 |
| Li Z-J2021 | 20，7，10，22 |
| Li B2018 | 20 |
| Kang J2025 | 20，17，11 |
| Na S J2018 | 1，19，22 |
| Carelli S2023 | 20，7 |
| Allou N2018 | 20，12 |
| Yeo H J2020 | 37 |
| Lee E H2022 | 20，16，19，18，8 |
| Hao T2024 | 1,20 |
| Wang J2021 | 20,19,2,17,15,13 |
| Juthani B K2018 | 20,10 |
| Manerikar A2022 | 17,11 |
| Rodriguez-Goncer I2018 | 1 |
| Massart N2023 | 14,19,17,15,11 |
| Kutleša M2017 | 20,16 |
| Pinna S M2023 | 20,5 |
| Ko R-E2020 | 13 |
| Xu W2022 | 20 |
| Martos A V H2023 | 3 |
| Kuo L-P2023 | 1,17 |
| Wang J-R2020 | 7,18, 14 |
| Yang L2022 | 20,16,2,15 |
| Deng Q2024 | 20 |
| Wang L2023 | 20,7,18,11 |
| R.R. Ling2023 | 1,16,5 |
| Marcus J E2021 | 18 |
| Winiszewski H2022 | 20,17 |

NOTE: 1.Immunosuppression; 2.Gender ; 3.Transport; 4.Virus; 5.Comorbidities; 6.Infection; 7.Mechanical ventilation; 8.Duration to ECMO; 9.C-reactive protein; 10.Hospital Stay Days; 11.Lactate; 12.SAPS II; 13.Body Mass Index; 14.SOFA; 15.Hypertension; 16.CRRT; 17.Diabetes(T2DM); 18.Mode; 19.Age; 20.ECMO Duration; 21. Catheter Intubation; 22. Arterial Catheter
