# Supplementary material for: Risk factors for nosocomial infections in ECMO patients: a systematic review and meta-analysis
Source: Front Public Health. 2026 Jun 11;14:1820017. doi: 10.3389/fpubh.2026.1820017 (PMC13294189; doi:10.3389/fpubh.2026.1820017)
Supplement: Supplementary file 6 [file Table_3.docx]

Table S3.Summary of Infection Diagnosis Criteria and Major Pathogens.

| **Study** | **Infection Types & Diagnostic Criteria** | **Key Etiological Features** |
| --- | --- | --- |
| ****Aubron C**** | BSI, VAP, CAUTI – Modified CDC/NHSN [1]; ECMO window: >48h post-start to 72h post-stop. | G- dominant; VAP: Enterobacteriaceae 56.5%; BSI: Candida 37.5%, Enterococcus 20.8%. |
| ****Grasselli G**** | BSI, VAP, UTI, CRBSI, Cannula site infection – per ATS/IDSA & IDSA guidelines [2][3]. | G- 48%, G+ 35%, Fungi 17%; MDR in 56% of bacterial infections. |
| ****Bachleda T**** | Not defined. | No data. |
| ****Hsu M-S**** | Not specified. | G- 77.8% (E. coli dominant), G+ 16.7% (MRSA, VRE), Fungi 5.6%. |
| ****Sun H-Y**** | All HAI types – CDC [1]; ECMO window: cannulation to decannulation. | G- 68.8% (mainly S. maltophilia, P. aeruginosa, K. pneumoniae), G+ 16.7%, Fungi 14.6%. |
| ****Vogel A M**** | ELSO registry criteria [4] (new pathogen or WBC <1500/μL). | No data. |
| ****Schmidt M**** | VAP, BSI, Mediastinitis, Cannula site – CDC/NNIS [1]; VAP confirmed by BAL ≥10⁴ CFU/mL. ECMO window: 24h post-start to 48h post-weaning. | 222 episodes: VAP 55%, BSI 18%, Mediastinitis 11%, Cannula site 10%. |
| ****Bougle A**** | Not specified. | No data. |
| ****Kim G S**** | CDC [1]. | G- dominant (88.9% of respiratory, 66.7% of BSI); A. baumannii most common. |
| ****Li Z-J**** | Chinese Ministry of Health 2001 standard [5]; ECMO window: 24h post-surgery to 48h post-decannulation. | G- 84.9%, Fungi 15.1%, no G+; A. baumannii 28.3%, K. pneumoniae 15.1%. |
| ****Kang J**** | BSI: clinical + biomarkers (PCT, CRP) + mNGS. | mNGS-based; G- main pathogen (mainly A. baumannii). |
| ****Na S J**** | BSI: CDC-based [1] with primary/secondary classification; ECMO window >48h. | BSI: primary 19%, secondary 81% (source mostly respiratory); G- 38% of BSI. |
| ****Carelli S**** | BSI & VAP – international guidelines [2][6]; ECMO window 48h post-start to 48h post-end. | Primary BSI: G+ 88% (E. faecalis); Secondary BSI: G- 57% (A. baumannii/ P. aeruginosa). |
| ****Wang J-R**** | BSI, HAP – CDC-based [1]; HAP: >48h ECMO + sputum culture + two experts. | BSI: G- 73.7% (K. pneumoniae 31.5%); HAP: all G-. |
| ****Allou N**** | Cannula-related infection (CRI, custom): local signs + culture within 30 days. | Enterobacteriaceae 38%, Staphylococcus 28.2%, P. aeruginosa 18.3%; Fungi 1.4%. |
| ****Yeo H J**** | BSI, Catheter colonization, VAP – CDC/NHSN [1] & IDSA/ATS [3]; ECMO window: >48h to 1 day post-decannulation. | Control: mainly G+ (CoNS, Enterococcus), Candida spp.; Intervention: only Candida. |
| ****Lee E H**** | BSI: CDC-based [1]; window 12h post-cannulation to 7 days post-decannulation. | MRCoNS most common, VRE A. baumannii, K. pneumoniae; Candidemia: C. tropicalis predominant. |
| ****Hao T**** | VAP/BSI/UTI – CDC-like with quantitative cultures (VAP: BAL ≥10⁴, ETA ≥10⁵; UTI: ≥10³ CFU/mL). | G- 73.9%; A. baumannii in 62.5% VAP, K. pneumoniae in 57.1% BSI; MDR 41.3%. |
| ****Wang J**** | CDC-based [1] for BSI, respiratory, UTI, sternal wound. | G- 64.0%, G+ 27.8%, Fungi 8.2% (all Candida); A. baumannii most frequent. |
| ****Juthani B K**** | CDC [1] (>24h after admission). | G- 42.3%, G+ 34.6%, Fungi 23.1%. |
| ****Manerikar A**** | BSI (blood culture), catheter-related (local signs or tip culture). | G+ 42.1%, G- 31.6%, Fungi 26.3% (all Candida). |
| ****Rodriguez-Goncer I**** | Probable IPA (EORTC/MSG + culture/radiology). | No pathogen distribution. |
| ****Massart N**** | VAP & BSI – CDC-like, VAP confirmed by BAL ≥10⁴ or ETA ≥10⁵ CFU/mL. | VAP: non-fermenting G- 40.7%, Enterococcus 13.8%; BSI: Enterococcus 33.3%, Candida 16.7%. |
| ****Kutleša M**** | Hospital-acquired BSI: ≥2 sets for G+, 1 set for G- + SIRS, >48h after ICU admission. | G- 67.9% (A. baumannii 48.6% of all), G+ 28.6%; Fungi 3.6%. |
| ****Pinna S M**** | CDC-based [1]; VAP includes tracheobronchitis; CMV disease: DNA ≥1000 IU/mL. | G- 41.9%, G+ 25.8%, Viruses 12.9%; MDR 16.1%. |
| ****Ko R-E**** | Pneumonia (VAP), CRBSI, UTI, primary bacteremia, cannula site – IDSA & international guidelines [2][3]. | G- 60%, G+ 34.3%, Fungi 5.7%; in MDR infections G+ predominance. |
| ****Xu W**** | BSI, VAP, UTI – CDC/NHSN-like; VAP confirmed by semi-quantitative culture. | VAP/BSI: G- dominant (mainly A. baumannii, K. pneumoniae); MDR rate 85%. |
| ****Kuo L-P**** | VAP (fever + infiltrate + purulent sputum + culture ≥10⁶/10⁴ CFU/mL), BSI (2 independent cultures + signs). | G- 76.8% (P. aeruginosa, A. baumannii, K. pneumoniae), G+ 16.8%, Fungi 6.3%. |
| ****Yang L**** | BSI: CDC/NHSN [1] (2 independent cultures for same pathogen + signs). | BSI mainly G- (91.3%) (A. baumannii, B. cepacia, K. pneumoniae); MDR 34.8%. |
| ****Deng Q**** | HAI (VAP, BSI, CAUTI, CLABSI) – CDC 1988 [1]; window: 24h post-start to 48h post-end. | G- 71.6% (A. baumannii 31.9%), G+ & Fungi each 14.2%. |
| ****Wang L**** | Nosocomial infection – automated surveillance + clinician confirmation; window: 24h post-start to 48h post-weaning. | G- 85.0% (P. aeruginosa, A. baumannii, K. pneumoniae); Fungi mostly in UTI. |
| ****Marcus J E**** | Nosocomial infection (positive culture during ECMO/48h post, team-confirmed), by site. | MDR approximately 50% of evaluable bacterial infections. |
| ****Winiszewski H**** | Cannula-related infection: sepsis/local signs + local culture; bacteremia if blood culture matches. | Enterobacteriaceae 43.8%, Enterococcus 25.0%, CoNS 21.9%; no fungi. |
| ****R.R. Ling**** | Infection not site-defined. | G- 56%, no further breakdown. |

Notes:

[1] CDC/NHSN or CDC-based hospital-acquired infection definitions.

[2] ATS/IDSA 2005 guidelines for hospital-acquired/ventilator-associated pneumonia.

[3] IDSA 2009 guidelines for catheter-related BSI, UTI, etc.

[4] ELSO (Extracorporeal Life Support Organization) registry definitions.

[5] Chinese Ministry of Health “Hospital Infection Diagnostic Criteria (Trial)” 2001.

[6] Other international guidelines or custom modifications.
